# Overcoming resistance to EGFR monotherapy in HNSCC by identification and inhibition of individualized cancer processes

**DOI:** 10.7150/thno.64347

**Published:** 2022-01-01

**Authors:** Maria R. Jubran, Daniela Vilenski, Efrat Flashner-Abramson, Efraim Shnaider, Swetha Vasudevan, Ariel M. Rubinstein, Amichay Meirovitz, Shay Sharon, David Polak, Nataly Kravchenko-Balasha

**Affiliations:** 1 The Institute of Biomedical and Oral Research, Hebrew University of Jerusalem, Jerusalem 91120, Israel.; 2 Sharett Institute of Oncology, Hebrew University-Hadassah Medical Center, Jerusalem, Israel.; 3 Department of Oral and Maxillofacial Surgery, The Hebrew University and Hadassah Medical Center, Jerusalem, Israel.; 4 Department of Periodontics, Faculty of Dental Medicine, The Hebrew University and Hadassah Medical Center, Jerusalem, Israel.

**Keywords:** precision medicine, targeted therapy, head and neck squamous cell carcinoma, information-theoretic approach, patient-specific signaling signatures

## Abstract

Therapeutic strategies for advanced head and neck squamous carcinoma (HNSCC) consist of multimodal treatment, including Epidermal Growth Factor Receptor (EGFR) inhibition, immune-checkpoint inhibition, and radio (chemo) therapy. Although over 90% of HNSCC tumors overexpress EGFR, attempts to replace cytotoxic treatments with anti-EGFR agents have failed due to alternative signaling pathways and inter-tumor heterogeneity.

**Methods:** Using protein expression data obtained from hundreds of HNSCC tissues and cell lines we compute individualized signaling signatures using an information-theoretic approach. The approach maps each HNSCC malignancy according to the protein-protein network reorganization in every tumor. We show that each patient-specific signaling signature (PaSSS) includes several distinct altered signaling subnetworks. Based on the resolved PaSSSs we design personalized drug combinations.

**Results:** We show that simultaneous targeting of central hub proteins from each altered subnetwork is essential to selectively enhance the response of HNSCC tumors to anti-EGFR therapy and inhibit tumor growth. Furthermore, we demonstrate that the PaSSS-based drug combinations lead to induced expression of T cell markers and IFN-γ secretion, pointing to higher efficiency of the immune response.

**Conclusion:** The PaSSS-based approach advances our understanding of how individualized therapies should be tailored to HNSCC tumors.

## Introduction

Head and neck squamous cell carcinoma (HNSCC) is the sixth most common cancer worldwide 1. HNSCC patients demonstrate poor outcomes, with a 5-year overall survival rate of 40-50% 2,3.

As in many other types of cancer, also in HNSCC, selective Epidermal Growth Factor Receptor (EGFR) inhibitors (gefitinib and erlotinib) and EGFR antibodies (cetuximab) have demonstrated significant antitumor activity *in vitro* and *in vivo* 4. Cetuximab, a monoclonal antibody against EGFR, was approved for the treatment of primary and recurrent HNSCC, and is commonly administered to patients in conjunction with radiotherapy (RT) 2. However, it became shortly evident that the majority of patients do not respond to single-agent checkpoint blockade 5. Resistance to anti-EGFR monotherapy can arise due to different regulatory mechanisms that operate at the level of EGFR and its ligands, alternative parallel signaling pathways in the cells as well as the cross-talk between EGFR expressing cells and the tumor microenvironment 6. Recently, a new class of targeted immunotherapy - PD-1 inhibitors (nivolumab or pembrolizumab) were approved for the treatment of recurrent and metastatic, cisplatin-resistant HNSCC 5. It has been shown that blocking EGFR might also affect the mechanisms of resistance to immunotherapy 7, suggesting that the coordinated interruption of cooperative survival signaling pathways 8-10 in HNSCC 8,11,12 is necessary for optimal therapeutic results. However the promise for effective and selective drug combinations has been hindered by intensive inter-tumor heterogeneity of HNSCC 13,14. Therefore, novel approaches that will enable the design of individualized anti-cancer drug cocktails are vitally needed.

We suggest to improve the anti-HNSCC therapeutic strategies by resolving the individualized altered signaling structures in head and neck cancers. We implement an approach based on surprisal analysis (SA), a thermodynamic-based information-theoretic method 10,15-17, to identify a patient-specific set of altered signaling subnetworks, named unbalanced processes, in each cancer tissue 10,16. Based on those subnetworks 10 we assign a personalized treatment, comprising a combination of selective, anti-cancer targeted inhibitors. The approach was applied recently to melanoma and triple negative breast cancer (TNBC) tissues, showing promising results in-vivo 18,19. Using a dataset of over 1000 tumor tissues, including 203 HNSCC, we demonstrate that each tumor harbor a patient-specific set of 2-4 distinct unbalanced processes. Some of the processes did not include EGFR, therefore other central proteins are selected as central drug targets. We suggest that all processes found should be targeted simultaneously in order to reduce the total signaling flux within the cells. We experimentally validate the approach by demonstrating that the individualized anti-HNSCC targeted therapy, not only selectively inhibits tumor growth but also enhances the immune response of human Peripheral Blood Mononuclear Cells (PBMC) co-cultured-with tumor cells.

## Results

### EGFR expression variability in EGFR expressing cancers

To study proteomic alterations in head and neck cancers we obtained a proteomic dataset comprising 203 human HNSCC tumors from the TCGA database (The Cancer Genome Atlas) (**Table S1**). The tumors were profiled using RPPA approach 20. We included in the dataset tumors from 4 additional types of human cancer: glioblastoma (GBM; n = 203), lung adenocarcinoma (LUAD; n = 234), lung squamous cell carcinoma (LUSC; n = 192), and skin cutaneous melanoma (SKCM; n = 206) (1038 tumors total; **Table S1**). The addition of these tumor types served two purposes: (1) Enlarging the dataset allows identifying patient-specific altered signaling signatures with increased resolution, and (2) GBM, LUAD and LUSC are EGFR-overexpressing cancers, while SKCM tumors generally less express EGFR 21,22. Therefore, the analysis of these tumor types in conjunction with HNSCC may add important insights into the molecular processes underlying these tumors, particularly the EGFR-related processes.

We first examined the EGFR expression pattern in the 1038 tumors (**Figure S1A**). EGFR was overexpressed primarily in GBM and HNSCC, and to a lesser extent in LUAD and LUSC tumors (**Figure S1A**). SKCM tumors demonstrated only a slight variation in EGFR expression, and generally do not overexpress the receptor (**Figure S1A**). Looking at the expression levels of pY (1068) EGFR, the picture is more pronounced: GBM, HNSCC, LUAD and LUSC exhibit high levels of pY (1068) EGFR, while SKCM demonstrates less activity as reflected by pY (1068) EGFR levels (**Figure S1B**).

The analysis shown in Figure S1 corresponds to the previous findings stating that GBM, HNSCC, LUAD and LUSC are EGFR-expressing cancers, while SKCM less 21,22. When planning effective anti-cancer treatment regimens, however, such analyses do not provide accurate and patient-specific information. For example, 14 of the 203 HNSCC patients (6.9%) expressed EGFR levels that equal to the median value in the dataset or less (**Figure S1C**). 105 of them (51.7%) demonstrated EGFR expression levels of less than 1.5-fold of the median (**Figure S1C**). 8 of the 203 GBM patients (3.9%) were found to express EGFR levels that match the median value or less (**Figure S1C**). 40 of them (19.7%) express EGFR levels that equal 1.5-fold of the median value or less (**Figure S1C**). All these patients may benefit less from the anti-EGFR monotherapy, and may demand other modes of therapy. To predict which therapy, or combination of therapies, should elicit a favorable response in every tumor, it is essential to decipher the structure of the patient-specific altered signature 18.

### Calculation of patient-specific altered signaling signatures (PaSSS) in each tumor

**Figure 1:**
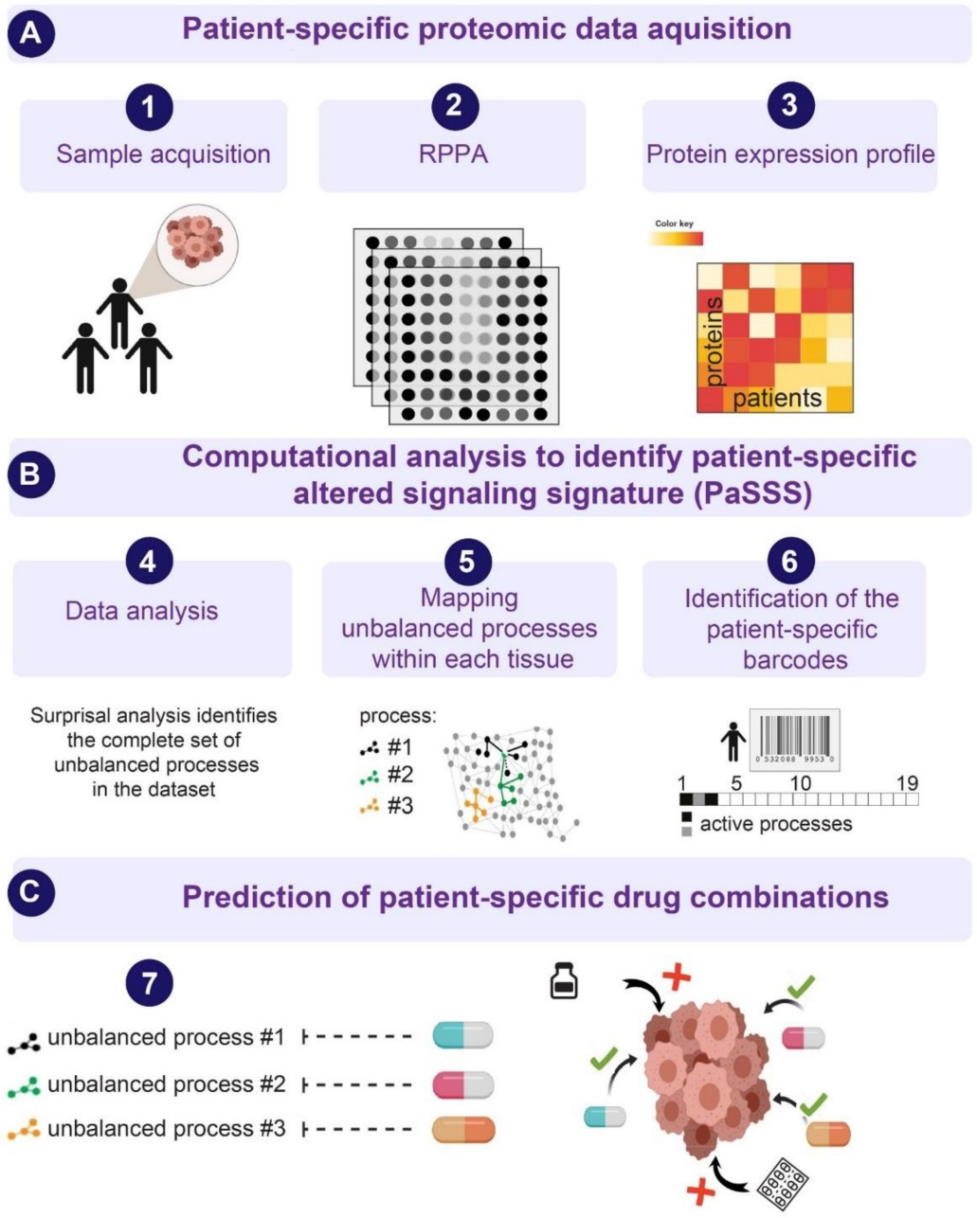
**An overview of the thermodynamic-based approach for calculation of patient-specific signaling signatures**. (**A**) Each cancer tissue is profiled for functional oncogenic proteins using, for example, Reverse Phase Protein Array (RPPA) approach. (**B**) Protein expression levels from each tissue are used as an input for surprisal analysis (SA). SA identifies active unbalanced processes in the population of cancer patients, which are utilized further to compute an altered, patient-specific signaling signature (PaSSS) in every sample, encompassing the individualized set of unbalanced molecular processes (i.e. groups of proteins that undergo deviations from their balanced expression levels in a correlated, function-related manner; see main text and Methods). (**C**) Based on the information obtained from each PaSSS, the response of the patient-specific protein networks to various conditions, including drug treatments, can be predicted. Consequently, combination therapies that should elicit an effective response in every tumor can be rationally designed. We hypothesize that at least one central hub protein from *every* unbalanced process should be targeted in order to efficiently collapse the entire imbalance in the specific tumor. This figure was created using BioRender.com.

Next, we examined HNSCC tumors by utilizing the SA-based approach 10,16,18,23, to study the patient-specific signaling signatures and explore additional therapeutic modalities for HNSCC (**Figure 1**). Briefly, using protein expression data (**Figure 1A**) the analysis identifies the signaling signature in each malignancy that may consist of several altered subnetworks, called unbalanced processes 10 (**Figure 1B**). Unbalanced processes are groups of proteins that display coordinated aberrations in expression levels and are thus presumed to have deviated from their balanced levels due to a common perturbation on the system 15 (**Figure 1B, Materials and Methods**). Each process is assigned an amplitude (weight) reflecting the importance or extent of activity of the process in each patient. Only processes with significant amplitudes, those that exceed error limits 16 are assembled into patient-specific sets of processes 10,18 (**Figure 1B**). These personalized sets of unbalanced processes are transformed schematically into patient-specific barcodes (**Figure 1B,** right panel). Deciphering the complete set of unbalanced processes (namely, the patient-specific altered signaling signature, PaSSS) in every patient, along with the central targets representing each process, enables the prediction of effective targeted therapies, many of which already exist in clinics (**Figure 1C,** 10). We hypothesize that it is essential to target at least one key protein from *every* unbalanced process to collapse the entire altered signaling flux 18,19 and inhibit the growth of the HNSCC tumors.

The PaSSS-based analysis of 1038 tumors revealed that 19 unbalanced processes repeat themselves throughout the dataset (**Figure S2A**). A rigorous error analysis was performed to find the accurate number of the processes characterizing this dataset and their protein composition (16 and **Figure S3**). We found that every tumor is characterized by a specific subset of these 19 processes, typically 2-4 processes in each barcode (**Table S2**).

Although pEGFR was found to participate in 9 different processes (**Figure S2A**) characterizing the EGFR overexpressing cancers, the analysis revealed additional ten processes in which pEGFR was not found to participate. Moreover, pEGFR+ processes demonstrate marked differences. For example, in unbalanced process 2 pY (1068) EGFR is correlated with pY (1248) HER2 and VEGFR2, and anti-correlated with pS (473) Akt, whereas in unbalanced process 5 pY (1068) EGFR is correlated with GAPDH and anti-correlated with HER2. Thus, measuring the overall changes in expression levels of several biomarkers, without accurate mapping within the altered *sub*networks, may overlook their relationships with other proteins in the altered network and therefore the essential information regarding the rational design of therapy combinations. We suggest that only the knowledge on the individualized structures of altered signaling can provide the relevant information for the effective design of anti-HNSCC treatments.

### PaSSS analysis divides 1038 tumors into 261 cancer subgroups

To compare the patients in the dataset to one another, we grouped the patients with identical barcodes into subgroups. We found 261 unique barcodes, reflecting patient-specific signaling signatures, that repeated themselves in the 1038 tumors tested (**Table S3**). Note that two tumors harboring the same barcode carry similar aberrations in the molecular processes and are therefore expected to respond to the same combination of drugs. Therefore, 261 distinct barcodes imply that the 1038 tumors can be divided into 261 subgroups of tumors. Interestingly, only 19 of the barcodes (indexed 1-19 in **Table S3**) each represent more than 10 tumors (~1% of the dataset). The remaining *242* barcodes (indexed 20-261 in **Table S3**) each characterize very small groups of tumors, each encompassing less than 10 tumors. Furthermore, 173 of the barcodes (**Table S3**) each characterize only a *single* tumor.

Significantly, the groups of tumors with identical barcodes did not necessarily contain tumors of the similar type: patients harboring tumors of the same type were found to be characterized by various barcodes, and vice versa - a certain barcode could characterize patients bearing tumors from various origins (**Table S3**). This finding underscores the importance of analyzing tumor data in an unbiased manner that depends only on the specific molecular aberrations that emerged in each tumor.

**Figure 2:**
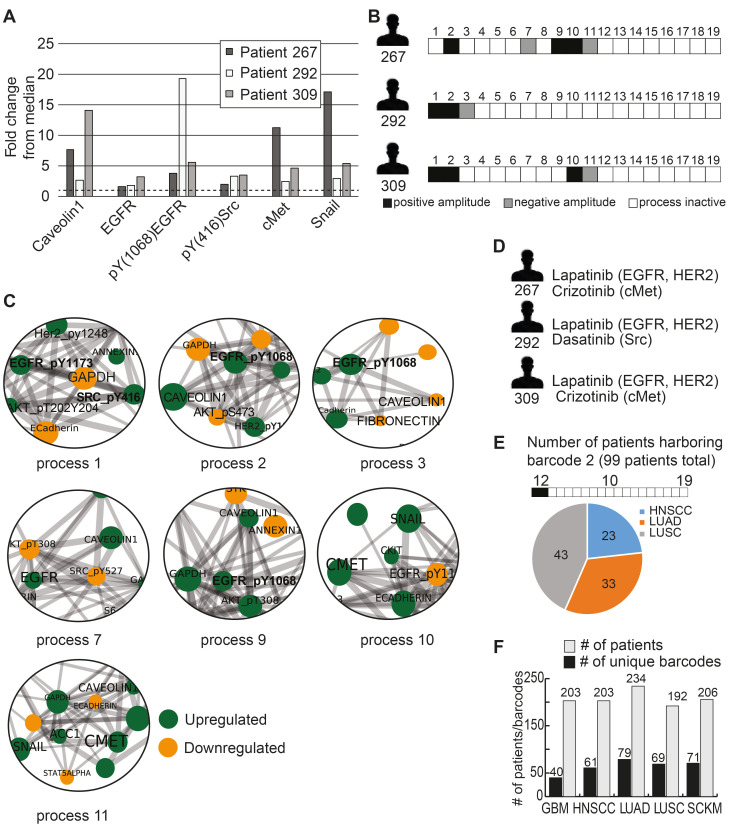
**Different patients with similar biomarker expression levels may harbor biologically distinct tumors**. (**A**) Three HNSCC patients were selected to demonstrate this point: patient 267, patient 292 and patient 309. The expression levels of 6 HNSCC-related protein biomarkers were examined, showing that in all three patients these biomarkers were upregulated relative to their median values. The dashed line in the graph marks the x = 1 level. (**B**) However, PaSSS analysis revealed that these patients have different barcodes as they harbor different sets of unbalanced processes. (**C**) Zoom in images of the unbalanced processes 1,2,3,7,9,10 and 11, which characterize patients 267, 292 and 309, and the participation of the 6 HNSCC-related biomarkers in those processes, are presented. To determine the direction of change in every protein (i.e. upregulation or downregulation due to the process) the amplitudes of the processes in these patients were considered. Note that in other patients the directions of change may be opposite. See Methods for more details. The complete set of unbalanced processes is presented in Figure S2. (**D**) Drug combination prediction for patients 267, 292 and 309 based on their unbalanced processes. (**E**) 99 patients out of 1038 were found to harbor *barcode* 2, in which processes 1 and 2 are active. These 99 patients encompass 23 HNSCC patients, 33 LUAD patients, and 43 LUSC patients. (**F**) The graph represents the uniquely characterized tumors along with the total number of patients in each cancer type; e.g. 61 barcodes, representing different altered signaling signatures, were identified in 203 HNSCC patients.

To demonstrate this, we selected 3 HNSCC patients: patient 267, patient 292 and patient 309. These patients all demonstrate upregulation of HNSCC-associated protein biomarkers (relative to the median expression levels of each protein): caveolin 1 24, EGFR 25, pY (1068) EGFR 25, pY(416)Src 26, cMet 25, and Snail 27 (**Figure 2A**). According to this list of protein biomarkers, these tumors may be defined as similar for the purposes of diagnostics and treatment. However, PaSSS analysis revealed that these patients harbor different signaling signatures, and they were therefore assigned different barcodes (**Figure 2B**). In all 3 patients, pEGFR was highly induced, a finding that is likely to lead clinicians to suggest EGFR inhibitor (e.g. cetuximab/erlotinib) as a treatment modality for all 3 patients. PaSSS analysis reveals different results: for example, PaSSS in patient 309 included 4 unbalanced processes, 2 of them (#1, 2) included induced pEGFR while other 2 processes (#10, 11) had induced cMet as a central target (**Figure 2B,C**). Thus EGFR inhibition is not expected to reduce the entire signaling flux in this patient. Cancer resistance may emanate from untargeted subnetworks. Thus combined treatment for this patient should include EGFR/HER2 and cMet inhibitors (**Figure 2D**). Similarly, patient 267 will require at least 2 different drugs (anti-EGFR and anti cMET) to reduce the signaling flux (**Figure 2B-D**). pSrc inhibition from the process 1 can be suggested as an additional treatment (for example, to enhance the efficacy of anti-EGFR/HER2 therapy in patient 292, with exceptionally high expression levels of pEGFR).

On the other hand, there are barcodes that may characterize several types of tumors. For example, barcode 2 (**Table S3**), the second most significant barcode, was found to characterize 99 of the 1038 patients (9.5%; **Figure 2E**). These 99 patients harbored 3 types of tumors: HNSCC (**23** patients), LUAD (33 patients), and LUSC (43 patients) (**Figure 2E, Table S3**). These 99 patients harbor identical tumors in terms of the altered molecular processes they possess and should therefore be treated in a similar manner. **Table S4** lists suggested combined treatments for each patient based on PaSSS and available, FDA-approved anti-cancer drugs 28. In general, once PaSSS is determined, a clinician can select the specific drug targets based on practical considerations, such as inhibitor availability, drug costs, toxicity and drug interactions in the combined treatment. Moreover in certain cases more than one drug may be required in order to inhibit a highly active process within a certain PaSSS 18.

In general, each cancer type was characterized by multiple barcodes and demonstrated relatively high levels of heterogeneity (**Figure 2F**). For example, the 203 HNSCC patients contain 61 subgroups of distinct tumors (**Figure 2F**), with the largest subgroup harboring 24 patients (11.8% of the HNSCC patients; **Table S3**); the 234 LUAD patients were divided into 79 subgroups (**Figure 2F**), with the largest subgroup containing 38 patients (16.2% of the LUAD patients; **Table S3**). Figure 2F presents this information for GBM, LUSC and SKCM as well. The list of patient-specific barcodes can be found in Table S2.

### Tissues from the same HNSCC anatomical regions may harbor different barcodes

The dataset included 116 HNSCC samples, for which tumor location was recorded, from 4 different regions: oral cavity, oropharynx, larynx, and hypopharynx. We asked whether samples obtained from the same anatomical region demonstrated a certain similarity in terms of barcodes. 79 of the HNSCC patients harbored tumors in the oral cavity. These patients were characterized by 32 *distinct* barcodes. 31 of the HNSCC patients harbored tumors in the larynx and were characterized by 20 barcodes. 5 of the HNSCC patients harbored tumors in the oropharynx and they all had *unique* characterization (**Table S5**). Interestingly, barcodes 1-3 repeated themselves in at least 10 patients and could be found in 3 out of 4 anatomical regions. For example, barcode #1 harbored processes 2 and 5; and was found in 17 patients: 12 patients with cancer in the oral cavity, 4 patients in the larynx, and 1 patient with cancer in the hypopharynx. Several more barcodes could be found in at least two anatomical regions (e.g. barcodes #4,5 and 7), whereas others, barcodes #17-43, were unique and could be found only in 1 single patient. This data indicates that on the one hand different regions can be treated with similar combinations, but on the other we need to be cautious in assigning drugs to patients with the tumors from the same region, because they might have completely different signatures (see for example barcodes 17-43).

### PaSSS analysis enables the prediction of drug combinations for HNSCC *in vitro*

To validate the approach experimentally, we moved to analyze a proteomic data set obtained from known ATCC cell lines 20, which can be grown easily and manipulated in the laboratory. Using the PaSSS-based analysis we have analyzed a dataset comprising 41 HNSCC cell lines profiled for ~237 functional proteins using RPPA measurements. To enhance the accuracy of the analysis we added 2 additional types of human cancer: lung cancer (n = 93) and uterus cancer (n = 28) (Table S6) to receive a final dataset including 162 cell lines. Drug combinations were assigned for each cell line as described above.

Surprisal analysis revealed that 20 unbalanced processes were requested to characterize 162 cell lines (**Figure S4A-C**). Each cell line was assigned an individualized set comprised of 2-5 processes (**Table S7**). pY (1068) EGFR was found active in 8 different processes (**Figure S4A**).

To choose a drug combination for each cell line, we have computationally assigned a barcode to each cell line, in the same manner as described above. We found 104 unique barcodes that repeated themselves in the population of 162 cell lines tested (**Table S8**), indicating that those 162 malignancies can be divided into 104 subgroups of tumors. Interestingly, only 3 of the barcodes (indexed 1-3 in **Table S8**) each represented more than 5 malignancies. The remaining 16 barcodes (indexed 4-19 in **Table S8**) each characterized very small groups of malignancies, each containing less than 5 tumors. Furthermore, 85 of the barcodes (indexed 20-104 in **Table S8**) each characterize only a *single* cell line.

Although RPPA protein lists and cancer types, included in the patient-derived and cell line datasets, were different we could find similarities between these two datasets. For example, unbalanced process 3 from the cell line dataset (**Figure S4**) was comparable to the unbalanced process 2 from the patient-derived data set (**Figure S2**). For instance E-cadherin, pEGFR, pHER2 and EPPK1 were co-expressed and anti-correlated with PKCα (**Figures S2, S4**). pEGFR appeared as a central target in multiple processes: in patient-derived dataset (**Figure S2**) it was found in 9 different processes and in the cell line dataset it was found in 8 processes (**Figure S4**). Another example includes pS6, which appeared as a hub protein as well. It was involved in 6 processes in the tissue derived dataset and in 7 processes in the cell line dataset. Moreover, only in 3 processes pEGFR and pS6 were co-expressed. In others, they appeared independently.

### Experimental validation of PaSSS therapies

**Figure 3:**
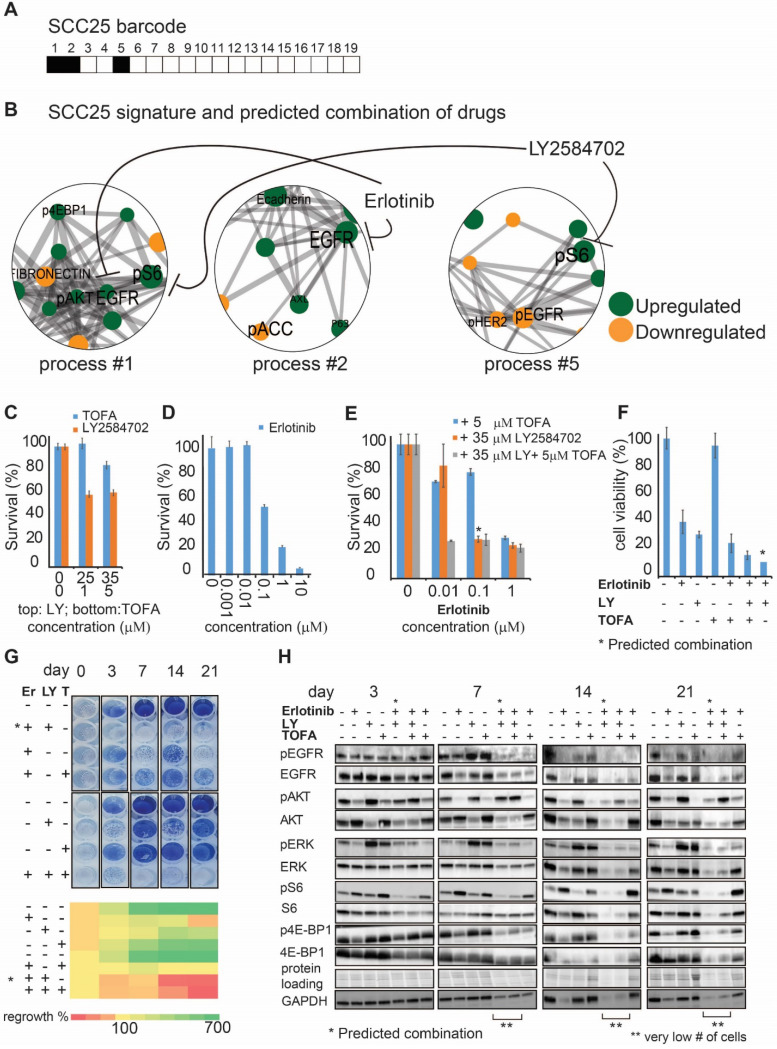
**The SA-based calculation of PaSSS allowed designing the efficient drug combinations for SCC25.** (**A, B**) Barcode, depicting the unbalanced processes and its emerging altered signaling signature in SCC25 cells, according to PaSSS analysis. Zoom-in images of the unbalanced processes active in SCC25 cells are shown (Figure S4 shows all participating proteins in each process). Correspondingly, the upregulation or downregulation of every protein is indicated in green or yellow, respectively. The complete number of unbalanced processes found in the cell line dataset is presented in Figure S4. The predicted drug combination and the processes each drug targets are shown (**B**). (**C, D, E**) Survival of SCC25 in response to different treatments and dosages. The combination of drugs predicted to target the unbalanced signaling signature (marked with an asterisk), as well as combinations that were predicted to partially target the unbalanced signaling flux of SCC25, were tested. (**F**) MTT assay confirms further the efficiency of the predicted drug combination (erlotinib and LY2584702). The predicted drug combination for SCC25 depletes the signaling flux and prevents cell regrowth. (**G**, upper panel) Cells were treated every three days and regrowth was measured up to 21 days with either monotherapy (erlotinib 0.1μM (**Er**), LY2584702 (**LY**) 35μM, TOFA (**T**) 5μM), the predicted drug combination (marked with an asterisk) or with the combinations that were predicted to partially target the unbalanced signaling flux of SCC25. Representative methylene blue-stained cell cultures are shown. (**G**, lower panel) Regrowth was quantified and presented as a heatmap. Day 0 represents the amount of cells seeded at the same day and was defined as 100%. Fold change relative to the amount of cells at day 0 is presented. Green color indicates higher regrowth levels (> 100%) and red shows decrease or depletion of the cells (< 100%). (**H**) Western blot analysis of the treated cells at different time points. The predicted drug combination is marked with an asterisk (*).

As a next step we selected two, EGFR expressing, oral tongue squamous cell carcinoma (OSCC) cell lines: Cal27 and SCC25 (Methods) from the cell line dataset in order to demonstrate experimentally the efficiency of the predicted drug combinations. Barcodes for those cell lines were calculated as described above and in Methods and can be found in **Table S7**. pS6 and EGFR proteins were identified as the main protein drug targets in SCC25 (**Figure 3A, B**), which were co-expressed in the process 1, but appeared as independent targets in two other SCC25 processes, processes 2 and 5, similarly to the tissue derived dataset. Thus, we suggested that both targets should be inhibited in order to reduce the unbalanced signature of SCC25 (**Figure 3A, B**). Based on this PaSSS, erlotinib (anti-EGFR) should be combined with LY2584702 (LY, anti-S6/S6K), to kill SCC25 cells efficiently and collapse the entire signaling network.

Whereas each drug alone, for example 35 µM LY (**Figure 3C**) and 0.1 μM of erlotinib (**Figure 3D),** killed around 40-60% of the cells, the predicted combination (*) brought about ~80% of cell death (**Figure 3E**). 5μM TOFA (Acetyl-CoA carboxylase (ACC) inhibitor), which was added to erlotinib, was significantly less effective than LY (**Figure 3E**), and did not increase the rate of cell death when was added to the combination of erlotinib and LY. ACC indeed was not found to participate in the signaling imbalance of SCC25 (**Figure 3B**). Additional survival assay (MTT, assessing metabolic activity of the cells) revealed the same result verifying further the efficiency of the predicted drug combination in comparison with other treatments (**Figure 3F**).

Furthermore, the predicted drug combination was significantly more efficient than other combinations or each drug alone in preventing cellular regrowth (**Figure 3G**). Analysis of the signaling proteins, related to the EGFR and S6 signaling pathways, revealed a gradual decrease in signaling activity during 3 weeks when the predicted combination was applied, until the complete cell death (**Figure 3H**). Anti-EGFR monotherapy failed to reduce pS6, whereas anti-pS6 was not efficient in reducing the EGFR related signaling (**Figure 3H**). This result points to the independent activity of those pathways and corresponds to the computational interpretation suggesting that EGFR and S6 proteins are involved in different processes and thus should be inhibited simultaneously. TOFA+erlotinib or TOFA+erlotinib+LY combinations were less efficient in reducing the cellular signaling during the period of 3 weeks (**Figure 3H**).

**Figure 4:**
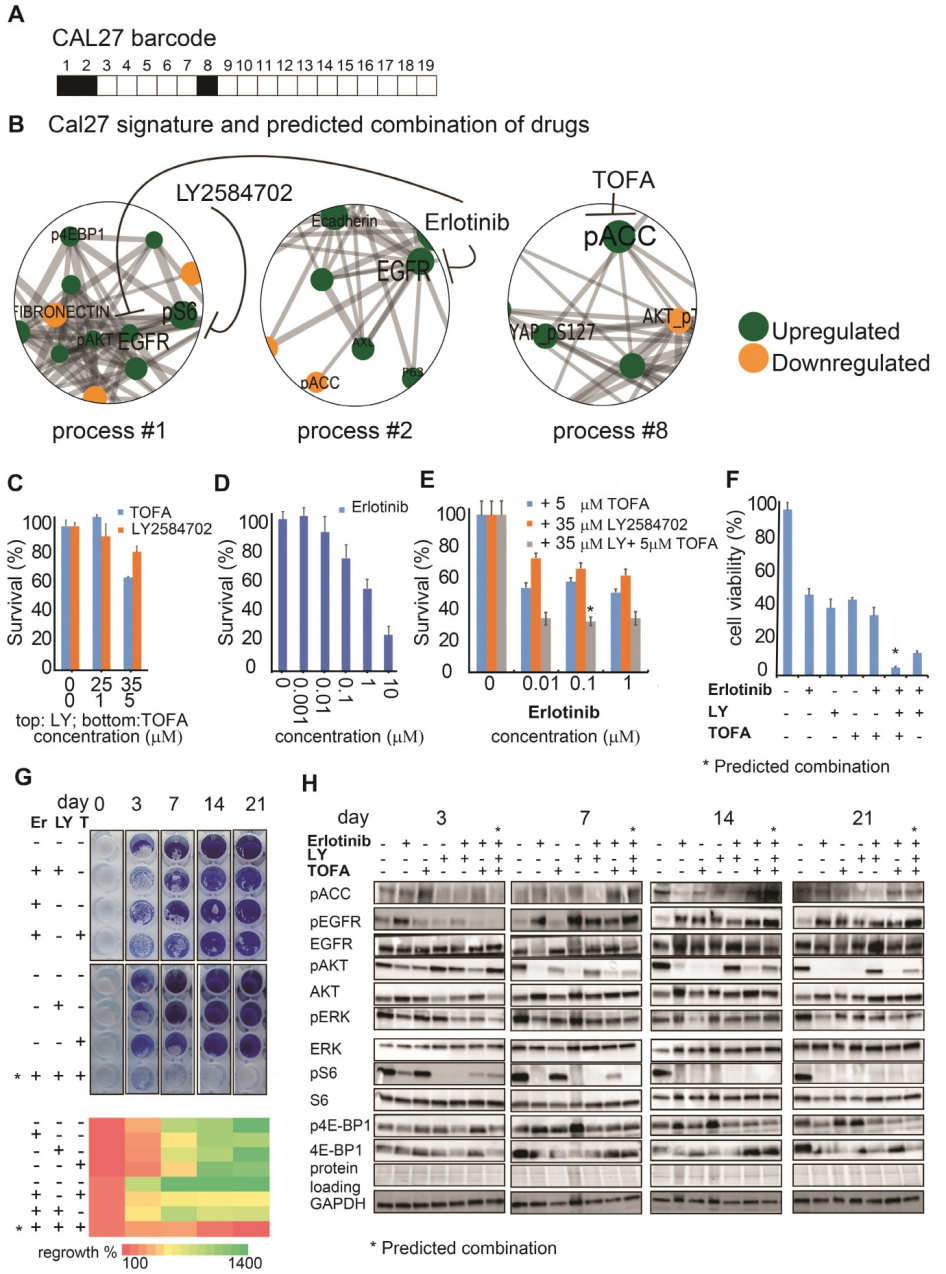
** The SA-based calculation of PaSSS allowed designing the efficient drug combinations for Cal27**. (**A, B**) Barcode, depicting the unbalanced processes and its emerging altered signaling signature in Cal27 cells, according to PaSSS analysis. Zoom-in images of the unbalanced processes active in Cal27 cells are shown (Figure S4 shows all participating proteins in each process). Correspondingly, the upregulation or downregulation of every protein is indicated in green or yellow, respectively. The predicted drug combination and the processes each drug targets are shown (**B**). (**C, D, E**) Survival of Cal27 in response to different treatments and dosages. The combination of drugs predicted to target the unbalanced signaling signature (marked with an asterisk), as well as combinations that were predicted to partially target the unbalanced signaling flux of Cal27, were tested. (**F**) MTT assay confirms further the efficiency of the predicted drug combination (erlotinib, LY2584702 and TOFA). The predicted drug combination for Cal27 depletes the signaling flux and prevents cell regrowth. (**G**, upper panel) Cells were treated every three days and regrowth was measured up to 21 days with either monotherapy (erlotinib 0.1μM (**Er**), LY2584702 (**LY**) 35μM, TOFA (**T**) 5μM), with the predicted drug combination (marked with an asterisk) or with the combinations that were predicted to partially target the unbalanced signaling flux of Cal27. Representative methylene blue-stained cell cultures are shown. (**G**, lower panel) Regrowth was quantified and presented as a heatmap. Day 0 represents the amount of cells seeded at the same day and was defined as 100%. Fold change relative to the amount of cells at day 0 is presented. Green color indicates higher regrowth levels (> 100%) and red shows decrease or depletion of the cells (< 100%). (**H**) Western blot analysis of the treated cells at different time points. The predicted drug combination is marked with an asterisk (*).

The predicted drug combination for Cal27 included 3 different drugs: TOFA, LY and erlotinib, as suggested by Cal27 signaling signature (**Figure 4A,B**). Experimental validation indeed confirmed that either monotherapies or double therapy (erlotinib and LY, which was efficient for SCC25) were significantly less efficient in killing Cal27 (**Figure 4C-E**).

Adding TOFA to erlotinib and LY significantly reduced the cell survival of Cal27 in opposite to SCC25, in which TOFA was ineffective. MTT assay confirmed further the efficiency of TOFA, LY and erlotinib combination (**Figure 4F**).

Moreover only the PaSSS-based combination of the 3 drugs prevented cellular regrowth in opposite to mono- or double therapies (**Figure 4G**). This combination brought about depletion of the cellular signaling in all tested proteins besides EGFR. Phosphorylation levels of EGFR were reduced after 3 days, but were induced again after 7, 14 and 21 days (**Figure 4H**). The long term activation of EGFR in response to erlotinib (e.g. measured after several days) corresponds to the studies by others (see for example 29. However, this activation was not sufficient to activate its downstream signaling, as represented by pAkt/pERK/pS6/p4E-BP1 levels, and initiate cellular regrowth of Cal27 cells, as a very low number of cells could be found after 21 days of treatment with the PaSSS-based combination (**Figure 4H**).

These results confirm the ability of the presented approach to determine the patient-specific signaling signatures with high accuracy and therefore to predict the individualized drug optimizations of anti-EGFR monotherapies. The results provide also a clear evidence that although the malignancies can be of the same cancer type (e.g. oral cancer from tongue), they may acquire different signaling reorganizations and thus require different individualized treatments.

### Patient-specific targeted therapy enhances CD8+ T cell activation potential

**Figure 5:**
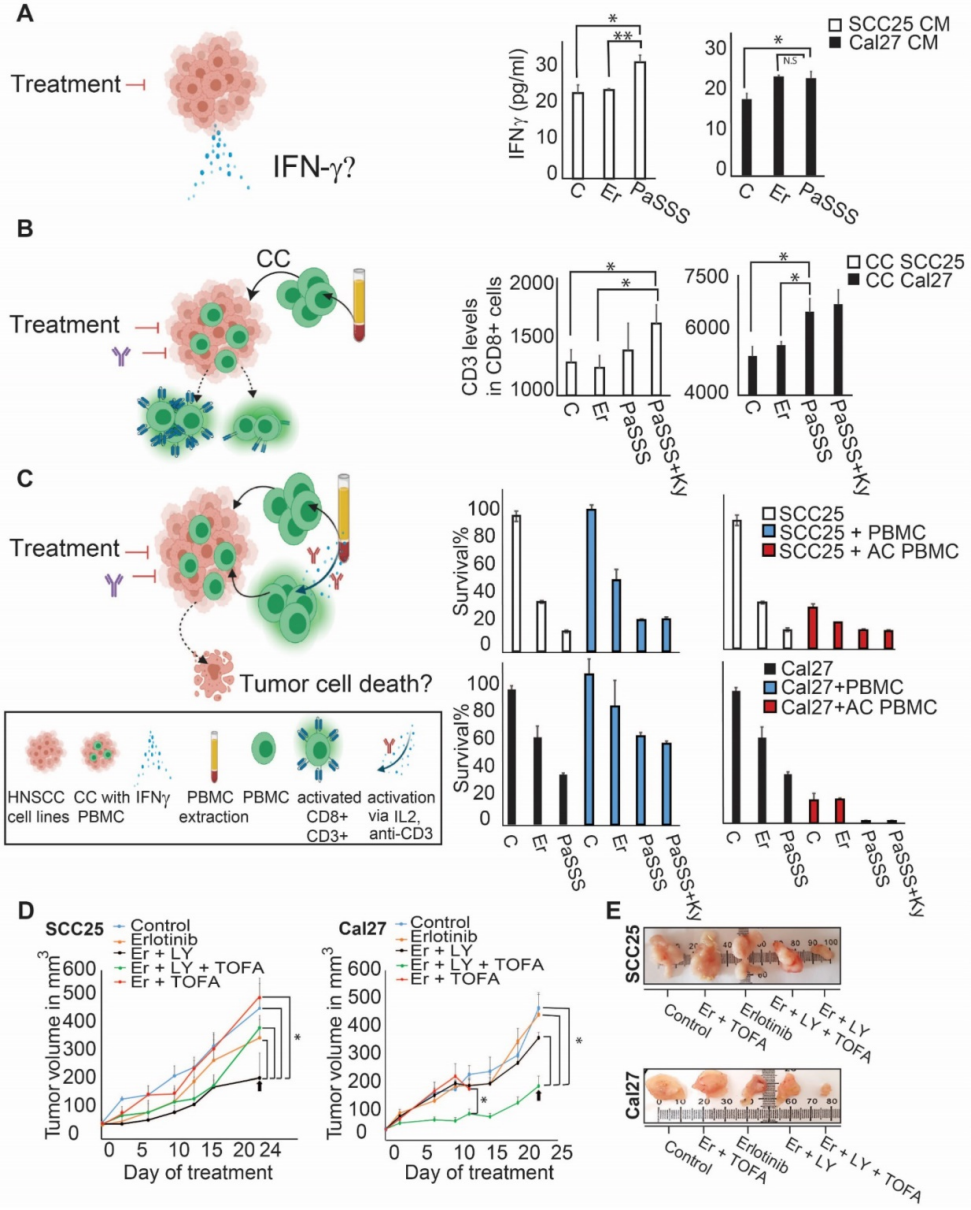
** The PaSSS-based drug combinations induced an immune response of PBMC in SCC25 and Cal27 models *in vitro* and reduced tumor growth *in vivo*.** (**A**) To examine IFN-γ secretion (as illustrated in the scheme on the left) SCC25 cells (middle panel) and Cal27 cells (right panel) were treated with either anti-EGFR monotherapy (**Er**) or the PaSSS-based combination. **C** stands for control. After 48h and 96h respectively the supernatants were collected for IFN-γ levels quantification (*P < 0.05, **P < 0.001). (**B**) SCC25 (middle panel) and Cal27 cells (right panel) were co-cultured (CC) with PBMCs (as illustrated in the scheme on the left) and treated for 48h and 96h respectively with either anti -EGFR monotherapy or the predicted combination (with or without the addition of 10μg/ml Keytruda, **Ky**). PBMCs were then collected and CD3 levels in CD8 positive cells were measured (*P < 0.02 for SCC25,*P < 0.007 for Cal27). (**C**) SCC25 (**C**, upper right panel) and Cal27 (**C**, lower right panel) were CC with non-activated /activated PBMCs (**AC PBMC**) and then the cells were treated with either anti-EGFR monotherapy or the predicted combination with or without the addition of 10 μg/ml Keytruda for 96h. Cell survival was measured via methylene blue. **(D)** SCC25 (**D,** left panel) or Cal27 (**D**, right panel) were injected subcutaneously into mice, and once tumors reached 50 mm^3^, treatments were initiated. In both cases, the PaSSS-based drug combinations (see black arrows) inhibited tumor growth and demonstrated an effect superior to monotherapy of erlotinib or to the drug combinations predicted to partially target the PaSSS (*P < 0.03 for SCC25) (*P < 0.03 for Cal27) (see **Figures 3,4** for details regarding the altered signaling signatures and the PaSSS-based drug combination predictions). (**E**) Representative, treated and untreated SCC25 and Cal27 tumors, harvested after 25 days and 14 days respectively, are shown. Panels (A-C) were created using BioRender.com.

In the recent years immunotherapy became one of the major treatment modalities in HNSCC. Recent studies connect between EGFR activation and immunosuppression, suggesting an additional role for EGFR as a modulator of tumor microenvironment (7). To compare the effect of EGFR monotherapies to the predicted individualized targeted therapies on immune response, we treated HNSCC cell lines with either monotherapies, or combined therapies, and examined a change in the secreted levels of interferon gamma (IFN-γ) in response to different treatments (**Figure 5A**, left panel). The results show that the PaSSS-guided treatment of SCC25 induced IFN-γ secretion compared to the anti-EGFR monotherapy (**Figure 5A**, middle panel). Cal27 cells responded similarly when either the anti-EGFR monotherapy or the PaSSS-based therapy were administrated (**Figure 5A**, right panel).

Next, we co-cultured oral cancer cell lines, with peripheral blood mononuclear cells (PBMC) obtained from healthy donors (**Figure 5B**, left panel).

Examination of CD8^+^ T lymphocyte activation potential (by measuring CD3 expression levels) in naïve PBMC, co-cultured with SCC25 cells, demonstrated a significant increase in CD3 expression when the predicted drugs combined with Keytruda (anti-PD-1 inhibitor) were added to the co-culture (CC, **Figure 5B**, middle panel). This result was even stronger in Cal27 model, showing enhanced CD3 expression in CD8^+^ cells in response to the PaSSS-based combination (**Figure 5B,** right panel). Although in SCC25 malignancy, the PaSSS-based treatment enhanced the levels of CD3 in CD8+ T cells when it was supported by anti-PD-1 inhibitor, the combination predicted for Cal27 enhanced the CD8+ T cell activation potential in Cal27 model without Keytruda support. Overall, these results indicate that the PaSSS-based combinations not only induce tumor cell death, but may also activate immune response. It remains to validate this effect in-vivo using a specialized (humanized) immune-competent mice model.

To examine whether the killing effect of cancer cells, initially induced by the individualized treatments, increases more upon induction of the T cell activation, we quantified the viability of HNSCC cells co-cultured with PBMC (**Figure 5C**, left panel). Although PBMC, either non-activated or pre-activated, did not enhance killing of SCC25 cells, the predicted for Cal27 drug combination eliminated Cal27 cells completely when Cal27 were co-cultured with pre-activated PBMC (**Figure 5C**, middle and right panels). This result may point to the different regulatory and/or anti-apoptotic mechanisms, which might exist in certain HNSCC malignancies, or to the requirement of additional microenvironmental parameters needed for PBMC in order to enhance the cell death of SCC25.

### The PaSSS-based drug treatments were required to inhibit tumor growth in-vivo

In order to investigate the effect of the PaSSS-based drug combinations in murine mice models HNSCC cells (SCC25 or Cal27) were injected subcutaneously into immunodeficient NSG mice, and treatments were carried 6 times a week for up to 4 weeks (Figure 5D).

Figures 5D and 5E show that the PaSSS-based combinations inhibited the tumor growth in both cases, and were more efficient than anti-EGFR monotherapies. Moreover the PaSSS-based combinations were highly selective, as the predicted and efficient combination for SCC25 malignancy (**Figure 5D, E**) was significantly more effective in SCC25 than the combination predicted for Cal27 and vice versa.

These results highlight further the need for the design of personalized treatments for HNSCC based on individualized alterations in signaling networks.

### Anti-EGFR monotherapy fails to reduce the unbalanced flux in HNSCC human samples and in certain cases induces initially inactive unbalanced processes

**Figure 6:**
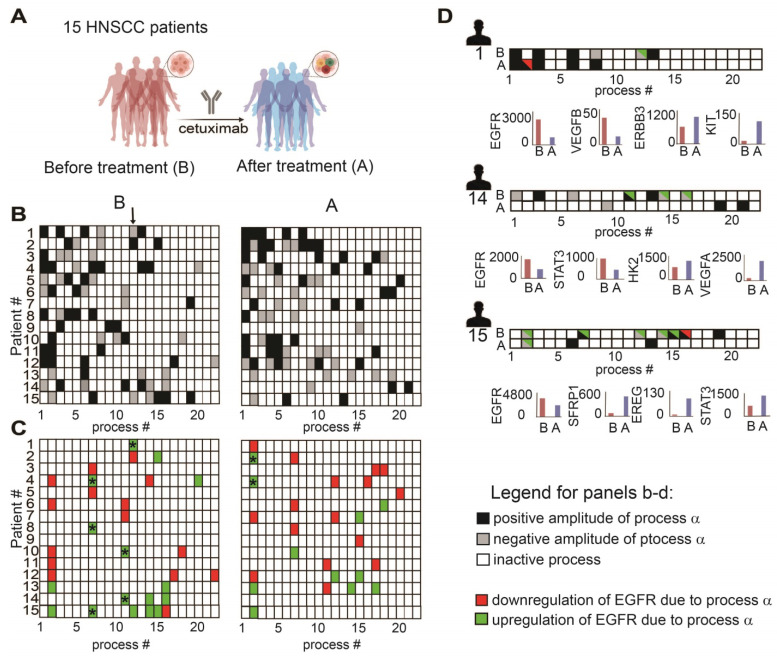
**Anti-EGFR monotherapy fails to reduce the unbalanced flux in HNSCC human samples.** (**A**) Changes in gene expression levels were acquired from 15 HNSCC patients before and after treatment with cetuximab for 2 weeks as shown in the illustration. (**B**) Patient-specific barcodes were generated for each patient before (left panel) and after the treatment (right panel). Negative/positive amplitude denotes how the patients are correlated with respect to a particular process. For example, patient 1 harbors process 12- (labeled with a black arrow), whereas patient 2 harbors process 12+. Therefore, transcripts that participate in process 12 (Table S9) deviate from the steady state in opposite directions in these patients. (**C**) Only processes in which EGFR participates are shown for each patient. EGFR upregulation or downregulation due to the process were defined as explained in the Figure legend of Figure S2 and labeled with green (upregulation due to a process) or red (downregulation due to a process) colors. In certain tumors, in which a clear reduction in the levels of EGFR and EGFR-related transcripts was detected, other onco-transcripts were upregulated. (**D**) Patient-specific barcodes are shown for patients #1,14,15. EGFR participation in active processes is labeled with green and red colors as indicated. Examples for a change in the experimental gene expression levels in response to EGFR inhibition are shown for selected genes and for each patient in lower panels. (B** -**barcodes before the treatment**;** A** -** after the treatment**)**. Panel (A) was created using BioRender.com.

To validate further our hypothesis, namely, that the anti-EGFR monotherapy should be optimized in a patient-specific manner, we analyzed changes in *gene* expression levels in response to cetuximab monotherapy (anti-EGFR) in *patient-derived* HNSCC tumors (GSE109756, GEO database). Using PaSSS analysis we analyzed tissues from 15 different patients who received cetuximab treatment for 2 weeks (**Figure 6A, B; Table S9 and Figure S5**). Certain processes with induced EGFR expression were abolished in response to treatment. For example process 12, the only process with EGFR in patient 1 before the treatment (**Figure 6C,** labeled with * in **Figure 6C, left panel**), disappeared in response to cetuximab (**Figure 6C, right panel**). This process included, for example, transcripts involved in cell adhesion and regulation of cell proliferation (**Table S10, Tab “G12 negative”**). Processes 7 and 11 (labeled with *) are additional examples for the EGFR+ processes which were reduced in response to treatment in almost all samples which harbored this process. However, many of the processes in which EGFR does not participate (shown in Fig. 6B), but also some EGFR+ processes, remained unchanged in response to treatment (**Figure 6B, C**). Moreover certain processes, for example EGFR+ process 2, were induced in patients 2 and 4 in response to cetuximab (**Figure 6C**, right panel, labeled with *). This process included different enriched categories related to cell proliferation, cell cycle and cell migration (**Table S10, Tab “G2 negative”**).

Figure 6D presents 3 different patient-derived tissues, as an example, in which a clear reduction of EGFR+ unbalanced processes was observed in response to cetuximab (**Figure 6D**). However, along with the profound reduction in the EGFR+ processes, other onco-processes were upregulated. For example in patient 1 (**Figure 6D**) process 2 appeared in response to the treatment, which corresponded to a decrease in EGFR levels, but an increase in the levels of cKit oncogene (**Figure 6D, patient 1, lower panel**; Table S10 includes additional genes/biological categories that characterize process 2). Similarly, an amplitude of process 1 was induced in response to the treatment (Table S9) leading to an increase in the gene expression levels of ERBB3 onco-receptor (**Figure 6D, patient 1, lower panel**).

Another two examples include patients 14 and 15 in which EGFR and the associated unbalanced processes were reduced by cetuximab, whereas other onco-transcripts and the processes as indicated in Figure 6D were induced. These results show that anti-EGFR monotherapies not only fail to reduce the entire tumor imbalance but can also induce various, previously inactive processes leading to reorganization of the network structures 19.

## Discussion

Assigning the right anti-cancer drugs to the right HNSCC patient is pivotal for generating positive treatment outcomes. Using the information-theoretic, SA-based computational approach 15,18,23, we provide a quantitative characterization of the inter-patient HNSCC heterogeneity in order to resolve patient-specific network reorganizations. We suggest that an accurate characterization of the individualized network alterations should contain information sufficient to predict the response of patient-specific networks to different therapeutic modalities.

Our study included a large dataset which was obtained from a cohort of 1038 tumors, encompassing 4 different cancer types and more than 200 HNSCC patients. Rather than relying on known signaling pathways, such as EGFR regulated pathways, PaSSS analysis identifies individualized groups of co-varying proteins, deviating in a similar manner from the reference state, in every tumor. Each such a group was defined as an unbalanced biological process.

We uncover the individualized* set* of unbalanced signaling processes in *every single patient*, namely patient-specific altered signaling signature (PaSSS). These processes are distinct, and therefore we hypothesized that each of them must be targeted individually in order to reduce the tumor-specific signaling imbalance 18,19. Importantly, PaSSS approach addresses individual patients in an unbiased manner rather than assigning them to pre-defined groups of patients (e.g. according to the status of EGFR or anatomical origin).

We show that 19 altered molecular processes capture the inter-tumor heterogeneity of 1038 tumors, where each tumor is characterized by a specific *subset* of 2-4 unbalanced processes. Accordingly, each HNSCC patient is assigned a unique barcode, denoting the patient-specific altered signaling signature. We found that the collection of 1038 tumors is described by 261 distinct barcodes. A subpopulation of 203 HNSCC patients is characterized by 61 unique barcodes, suggesting that the cohort of these patients consists of 61 subtypes of HNSCC cancer. Moreover we show that HNSCC tissues from different anatomic regions might have similar barcodes and thus should be treated with similar drug combinations, whereas patients harboring cancer in the same region might have completely different signaling signatures.

These 61 subtypes of HNSCC tumors, each representing a signaling barcode, are assigned patient-tailored combinations of drugs, many of which already exist in clinics for the treatment of different types of cancer. For example lapatinib (dual anti-EGFR and HER2 inhibitor), used for the treatment of breast cancer 28, appears frequently in PaSSS analysis as a suggested drug in mono- or combined treatments for HNSCC. Another example includes crizotinib (anti-cMet inhibitor, approved for the treatment of lung cancer) that appears in certain cases, usually in combination with lapatinib, as a suggested drug for HNSCC patients.

We demonstrate experimentally our ability to rationally design effective anti-HNSCC drug cocktails by analyzing a proteomic dataset including ATCC human cancer cell lines. We decipher the altered signaling signatures in these cell lines (PaSSS), and then design drug combinations that are predicted to target the unbalanced signaling flux in each sample. We used two HNSCC EGFR overexpressing cancer cell lines for experimental validation: SCC25 and Cal27. We demonstrate herein that by combining anti-EGFR (erlotinib) with one or two additional drugs, as predicted by the PaSSS-based analysis, the efficacy of the treatment enhances significantly. We show that although SCC25 and Cal27 belong to the same genetic subtype (e.g. EGFR overexpressing) and from the same anatomic region (e.g. tongue), different drug combinations were predicted to be most efficient for each of the malignancies. In both cases, our predicted drug cocktails demonstrated high potency and achieved higher rates of tumor inhibition than anti-EGFR monotherapy. Moreover the PaSSS-based drug combinations were highly selective. The efficient and predicted drug combination for SCC25 was less efficient for Cal27 and vice versa. Furthermore we demonstrate that the PaSSS-based drug combinations led to enhanced expression of CD8/CD3 T cell markers and IFN-γ secretion than anti-EGFR monotherapies, pointing to the enhanced ability of the patient-specific combinations to activate the immune system. This result suggests that the immune cells may further enhance the tumor response to the PaSSS-based combinations. Studies are underway in our laboratory, aiming to evaluate the efficiency of the PaSSS-based therapies in the presence of human immune system in humanized mice models. Moreover PaSSS therapy, which may be further combined with immunotherapy strategies, may provide long-term efficacy for HNSCC patients.

Analysis of the gene expression data obtained from HNSCC patients, treated with anti-EGFR monotherapies, validated further our hypothesis. We found that anti-EGFR monotherapy did not reduce efficiently the patient-specific molecular imbalance. Moreover we have shown that in certain cases anti-EGFR monotherapy led to the induction of new, previously undetected unbalanced processes, leading to a change in the signaling states of HNSCC patients. This result provides an additional support to the idea that in order to reduce the PaSSS-specific flux, anti-EGFR monotherapies should be replaced by patient-specific drug combinations, in which anti-EGFR drugs might be one of the suggested inhibitors in the individualized combined treatment 19.

In essence we suggest how a high complexity of the HNSCC tumors can be reduced to the simple, PaSSS-based, signaling barcodes that guide the rational design of patient-specific drug therapies. This deep understanding of the patient-specific signaling imbalances should advance the fields of personalized HNSCC research and therapeutics.

## Materials and Methods

### Surprisal analysis

Surprisal analysis, a thermodynamic-based information-theoretic approach, was applied in this study as detailed in 10,18. Briefly, the analysis is based on the premise that biological systems reach a balanced state when the system is free of constraints 15. However, when under the influence of environmental and genomic constraints, the system is prevented from reaching the state of minimal free energy. Each constraint can induce a change in a specific part of the protein network in the cells. The subnetwork that is altered due to the specific constraint is termed an unbalanced process. System can be influenced by several constraints thus leading to the emergence of several unbalanced processes. When tumor systems are characterized, the *specific set of unbalanced processes* is what constitutes the tumor-specific signaling signature (PaSSS).

Surprisal analysis discovers the complete set of constraints operating on the system in any given tumor, k, by utilizing the following equation 15: ln Xi(k) = ln Xi0(k) - ΣGiαλα(k), where *i* is the protein of interest, Xi0 is the expected expression level of the protein when the system is at the steady state and free of constraints, and ΣGiαλα(k) represents the sum of deviations in expression level of the protein *i* due to the various constraints.

The term Giα denotes the degree of participation of the protein *i* in the unbalanced process α. Proteins with significant Giα values (**Figure S2B,** 10,16) are grouped into unbalanced processes (**Figure S2A, S4A**) which are active in the dataset.

The term λα(k) represents the importance of the unbalanced process α in the tumor k. The detailed description on how Giα and λα(k) values are calculated can be found in the Supplementary file of the reference 15.

#### Determination of the number of unbalanced processes

To examine the number of significant processes in the dataset we check how many processes are required to reproduce the experimental data as described in **Figure S3** and 10,16,30. Threshold limits for *λ_α_(k)* were calculated as described previously 10,16,30.

#### Generation of functional subnetworks and barcodes representing PaSSSs

The functional subnetworks presented in Figures S2,S4 were generated by combining the Gi values (represented by the size of circles) and the String parametrs (used to generate functional maps) 16,18. The barcodes presented in Figures 2-4,6 and Tables S3, S7 were generated using *λ_α_(k)* values that exceeded the threshold limit in each sample *k*. The obtained patient-specific sets of *λ_α_(k)* values (unbalanced processes) were converted to -1 (for -*λ_α_(k)*), 0 (for insignificant *λ_α_(k)*) and 1 (for +*λ_α_(k)*) values 10,18.

#### For gene expression data

The procedure of SA was performed as described above. The biological meaning of the unbalanced processes was deciphered as following: transcripts with significant Gi values (Figure S5, 16) were grouped into biological categories according to Gene Ontology (GO) using David database (Table S10) as described previously 16.

### Selection of HNSCC cell lines for experimental validation of the PaSSS-Based Strategy

In this study we selected 2 EGFR expressing HNSCC cell lines SCC25 and Cal27, which were part of the cell line dataset (Table S6) to validate the approach. To provide a statistical meaning for the selection of 2 HNSCC cell lines we calculated an upper bound for the probability to select 2 and 3 unbalanced processes in HNSCC, as found in HNSCC cell lines, randomly. Calculation of the upper bound was based on the frequency of the most abundant unbalanced processes in the HNSCC subset calculated using a large set of 3467 tumors 10. The probability to find the three most abundant processes in a particular HNSCC malignancy equals to (*161*/212) × (*70*/212) × (*65/*212) = 0.076.

Numbers in italic represent numbers of HNSCC patients found to harbor the most abundant processes in the subset 10, e.g. processes 1, 2, and 3 and the number 212 is the number of HNSCC patients comprising this subset. The probability to find the two most abundant processes in a particular HNSCC sample was calculated in a similar manner and equals to (*161*/212) × (*70*/212) = 0.25.

Thus the upper bound for the probability to select 2 and 3 unbalanced processes, characterizing both, SCC25 and Cal27 malignancies randomly equals to 0.019.

### Cell Culture

The head and neck squamous cell carcinoma cell lines - SCC25 and Cal27 were obtained from ATCC and grown in DMEM (Cal27) or DMEM/F12 medium with sodium pyruvate (SCC25). They were supplemented with 10 % fetal calf serum (FCS), L-glutamine (2mM), 100 U/ml penicillin and 100 mg/ml streptomycin, and incubated at 37 ºC in 5% CO2. The cell lines were authenticated at the biomedical core facility of Technion, Haifa, Israel.

### Western blot analysis

The procedure was performed as described previuosly 31. The following antibodies were used: anti-pY (1068) EGFR (#3777), anti-PARP (#9542), anti-pS(235/236)S6 (#4858), anti- S6 (#2217), Phospho-4E-BP1 (Thr37/46) (236B4) (#2855), 4E-BP1 (53H11) (#9644), Phospho-Akt (Ser473) (D9E) XP, (#4060), Acetyl-CoA Carboxylase (C83B10)( #3676), Phospho-Acetyl-CoA Carboxylase (Ser79) (#3661), all were purchased from Cell Signaling Technology (Beverly, MA). Anti-ERK pT(202)Y(204) (E-4), Akt 1/2/3 (H-136), anti-EGFR(A-10), anti-ERK2 (c-14) and GAPDH (FL-335) were from Santa Cruz Biotechnology (Santa Cruz, CA). HRP-conjugated goat anti-mouse and HRP-conjugated goat anti-rabbit were from Jackson ImmunoResearch Laboratories, Inc. (West Grove, PA).

### Methylene blue assay

The assay was performed as described previously 10.

### MTT assay

The cells were seeded and treated as indicated in a 96 well plate for 72 hours. The cell viability was checked using MTT assay kit (Abcam). Equal volume of MTT solution and culture media was added to each well and incubated for 3 hours at 37 ºC. MTT solvent was added to each well, covered in aluminum foil and put on the orbital shaker for 15 minutes. Absorbance was read at 590nm within 1 hour.

### Resistance Assay

Cells were seeded in 96 well plates and treated as indicated for different time points (3, 7, 14, 21 days). At every time point the cells were fixed with 4% paraformaldehyde and quantified using methylene blue assay.

Following inhibitors were used for the above assays: erlotinib, LY2584702 were from Cayman Chemicals (Ann Arbor, MI), and TOFA was purchased from Abcam. The inhibitors were diluted in DMSO (equal concentration of DMSO was also used as a control in the appropriate experiments).

### Animal Studies

SCC25 (1 × 10^6^ cells/mouse) or Cal27 (1 × 10^6^ cells/mouse) were inoculated subcutaneously into NSG mice (n = 8 mice per group), and once the volume of the tumors reached 50 mm^3^, treatments were initiated 6 times a week for up to 4 weeks. Tumor volume was measured twice a week. erlotinib (15 mg/kg), LY (12.5 mg/kg) and TOFA (1 mg/kg) were suspended in aqueous mixture of 0.5% Hydroxypropyl methylcellulose + 0.2% Tween 80 and administered by oral gavage. LY-2584702 was purchused from the MCE (MedChemExpress).

The rest of the drugs were purchased from Cayman chemicals. The Hebrew University is an AAALAC International accredited institute. All experiments were conducted with approval from the Hebrew University Animal Care and Use Committee.

### Isolation of human PBMCs

PBMCs were collected from healthy volunteer (IRB approval number HMO-19-0033) using Polymorphprep™ (Axis-shield, Oslo, Norway) according to the manufacturer's instructions. Cells were cultured for 72h in 10 cm plate in complete RPMI-1640 media (RPMI-1640 medium supplemented with 10% FBS, 2 mM L-glutamine, 100 IU/ml penicillin and 100 µg/ml streptomycin and 0.1% 2-mercaptoethanol) at 37 °C in a humidified atmosphere containing 5% CO_2_.

When activated PBMC were used, their medium was also supplemented with purified human anti-CD3 (0.1 ng/ml) and IL2 (10 ng/ml) for 3 days incubation.

### Co-culture of PBMCs with HNSCC tumor cells

SCC25 or Cal27 cells were seeded at 10^5^ per well in their respective growth medium for 24h and then were co-cultured (CC) with equal number of naïve PBMCs or activated PBMC (preincubated with 0.1 ng/ml anti-CD3 and 10 ng/ml IL2 for 3days). Anti-EGFR monotherapy (0.1 μΜ erlotinib) or combined therapies (in which the following concentrations we re used: 35μM LY, 5μM TOFA, 0.1 μΜ erlotinib) as indicated with/without 10μg/ml Keytruda. After 48 and 96h of co-culture, the supernatants were collected for IFN-γ quantification using ELISA (R&D systems, Minneapolis, MN, USA) and PBMCs were collected for quantification of CD3 levels in CD8 cells using flow cytometry analysis (labeled with anti-CD45 (2D1/104), CD3, CD8 specific antibodies) using BD FACS LSR Fortessa.

### Statistical analysis

Statistical significance was determined by Student's t test (two tails, two samples equal variance); P values of ≤ 0.05 were considered statistically significant. All data represent the mean ± S.E. (standard error of the means). If not indicated otherwise, the experiments were performed at least three times.

### Availability of data and materials

The human tumor and cell line datasets that support the findings of this study can be found at TCPA portal 32, https://tcpaportal.org/tcpa/download.html. Gene expression data of HNSCC tissues treated with cetuximab can be found at GEO database, (GSE109756). Anti-cancer drugs used to design PaSSS combinations (Table S4) can be found in https://www.anticancerfund.org/en/cancerdrugs-db 28.

## Supplementary Material

Supplementary figures.

Supplementary table 1.

Supplementary table 2.

Supplementary table 3.

Supplementary table 4.

Supplementary table 5.

Supplementary table 6.

Supplementary table 7.

Supplementary table 8.

Supplementary table 9.

Supplementary table 10.
