# Supplementary material for: Overcoming resistance to EGFR monotherapy in HNSCC by identification and inhibition of individualized cancer processes
Source: Theranostics. 2022 Jan 1;12(3):1204–19. doi: 10.7150/thno.64347 (PMC8771558; doi:10.7150/thno.64347)
Supplement: Supplementary file 1 — Supplementary figures. [file thnov12p1204s1.pdf]

Supplementary Materials for

Overcoming resistance to EGFR monotherapy in HNSCC by identification and inhibition of individualized cancer processes

Maria R. Jubran[1], Daniela Vilenski[1], Efrat Flashner-Abramson[1], Efraim Shnaider[1], Swetha Vasudevan[1], Ariel M. Rubinstein[1], Amichay Meirovitz[2], Shay Sharon[3], David Polak[4]* and Nataly Kravchenko-Balasha[1]*

[1]The Institute of Biomedical and Oral Research, Hebrew University of Jerusalem, Jerusalem 91120, Israel

[2] Sharett Institute of Oncology, Hebrew University-Hadassah Medical Center, Jerusalem, Israel.

[3] Department of Oral and Maxillofacial Surgery, The Hebrew University and Hadassah Medical Center, Jerusalem, Israel.

[4] Department of Periodontics, Faculty of Dental Medicine, The Hebrew University and Hadassah Medical Center, Jerusalem, Israel.

*Corresponding authors: natalyk@ekmd.huji.ac.il, polak@mail.huji.ac.il

This PDF file includes:

1. **Figures S1 to S5**
2. **Supplementary References**

Figure S1

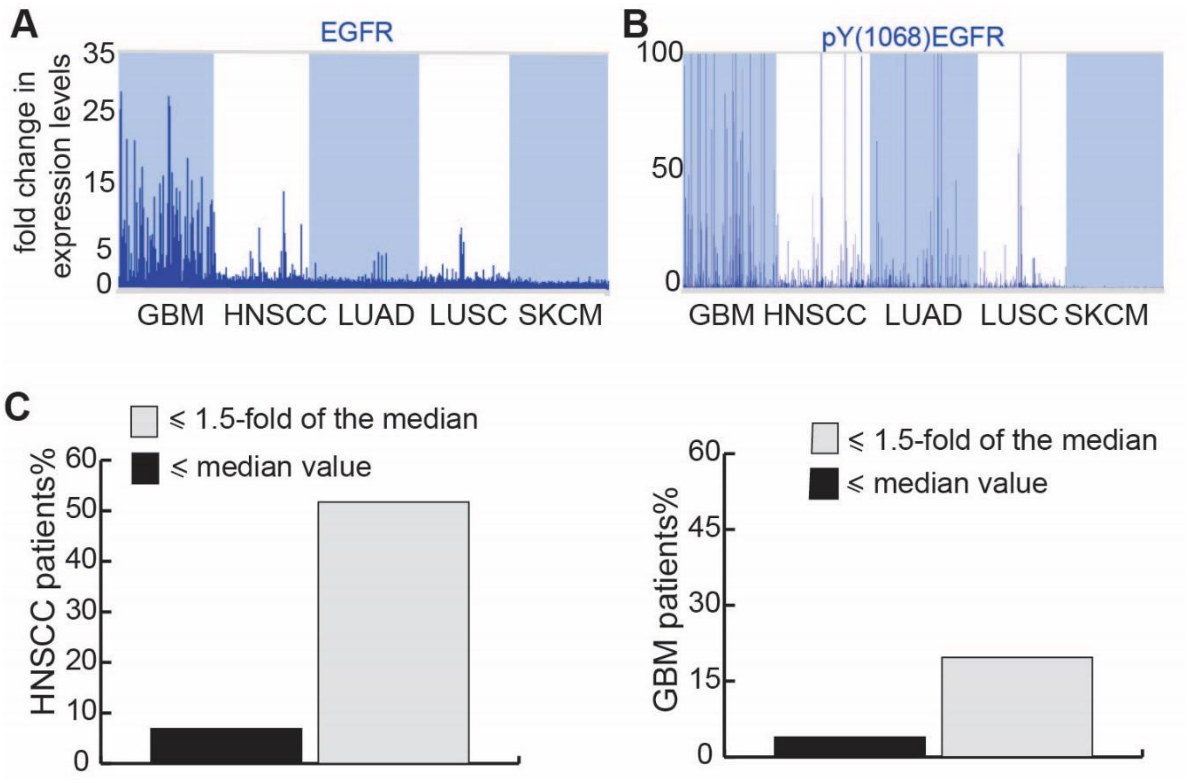

Figure S1. Variability in EGFR expression levels in different cancer types. EGFR expression pattern in 5 tumor types is presented. EGFR protein (**A**) and EGFR phospho-protein expression levels (**B**) are compared between GBM, HNSCC, LUAD, LUSC and SKCM cancer types and within each type. (**C**) Percentage of HNSCC and GBM patients that harbor relatively low EGFR levels in comparison with the median values in the dataset.

Figure S2

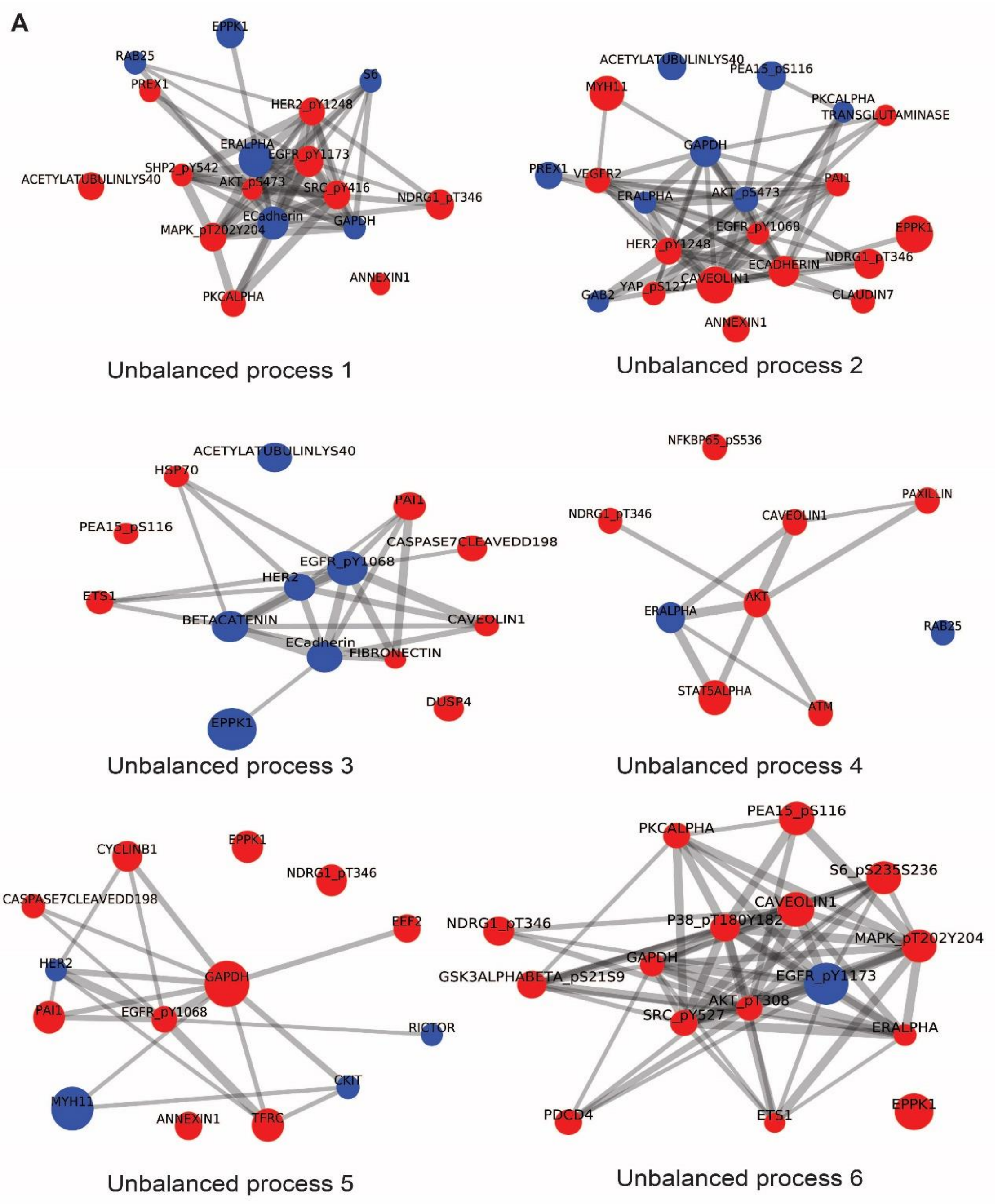
A
EPPK1
RAB25
S6
PREX1
HER2_pY1248
ERALPHA
EGFR_pY1173
SHP2_pY542
ACETYLATUBULINLYS40
AKT_pS473
SRC_pY416
NDRG1_pT346
ECadherin
GAPDH
MAPK_pT202Y204
ANNEXIN1
PKCALPHA
Unbalanced process 1
ACETYLATUBULINLYS40
PEA15_pS116
MYH11
PKCALPHA
TRANSGLUTAMINASE
GAPDH
PREX1
VEGFR2
PAI1
ERALPHA
AKT_pS473
EGFR_pY1068
EPPK1
HER2_pY1248
NDRG1_pT346
ECADHERIN
CAVEOLIN1
GAB2
YAP_pS127
CLAUDIN7
ANNEXIN1
Unbalanced process 2
ACETYLATUBULINLYS40
HSP70
PAI1
PEA15_pS116
CASPASE7CLEAVEDD198
EGFR_pY1068
HER2
ETS1
BETACATENIN
CAVEOLIN1
ECadherin
FIBRONECTIN
DUSP4
EPPK1
Unbalanced process 3
NFKBP65_pS536
PAXILLIN
NDRG1_pT346
CAVEOLIN1
AKT
ERALPHA
RAB25
STAT5ALPHA
ATM
Unbalanced process 4
CYCLINB1
EPPK1
NDRG1_pT346
CASPASE7CLEAVEDD198
EEF2
HER2
GAPDH
PAI1
EGFR_pY1068
RICTOR
CKIT
MYH11
ANNEXIN1
TFRC
Unbalanced process 5
PEA15_pS116
PKCALPHA
S6_pS235S236
CAVEOLIN1
NDRG1_pT346
P38_pT180Y182
MAPK_pT202Y204
GAPDH
GSK3ALPHABETA_pS21S9
EGFR_pY1173
AKT_pT308
SRC_pY527
ERALPHA
PDCD4
ETS1
EPPK1
Unbalanced process 6

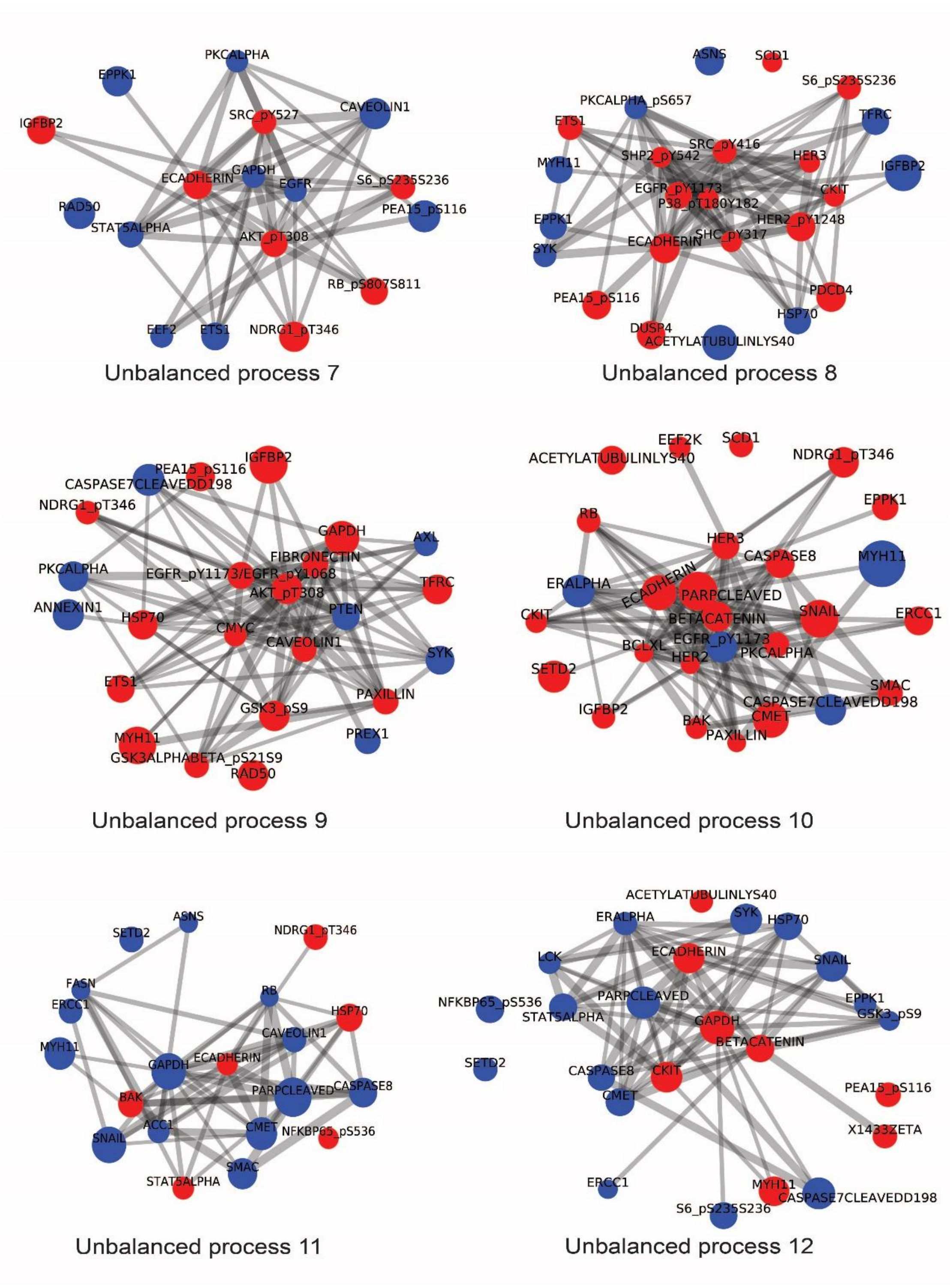
PKCALPHA
EPPK1
IGFBP2
SRC_pY527
CAVEOLIN1
ECADHERIN
GAPDH
EGFR
S6_pS235S236
RAD50
STAT5ALPHA
PEA15_pS116
AKT_pT308
RB_pS807S811
EEF2
ETS1
NDRG1_pT346
Unbalanced process 7
ASNS
SCD1
S6_pS235S236
PKCALPHA_pS657
ETS1
TFRC
SRC_pY416
SHP2_pY542
HER3
MYH11
IGFBP2
EGFR_pY1173
CKIT
P38_pT180Y182
EPPK1
HER2_pY1248
SHC_pY317
ECADHERIN
SYK
PDCD4
PEA15_pS116
HSP70
DUSP4
ACETYLATUBULINLYS40
Unbalanced process 8
IGFBP2
PEA15_pS116
CASPASE7CLEAVEDD198
NDRG1_pT346
GAPDH
AXL
FIBRONECTIN
PKCALPHA
EGFR_pY1173/EGFR_pY1068
TFRC
AKT_pT308
ANNEXIN1
PTEN
HSP70
CMYC
CAVEOLIN1
SYK
ETS1
PAXILLIN
GSK3_pS9
PREX1
MYH11
GSK3ALPHABETA_pS21S9
RAD50
Unbalanced process 9
EEF2K
SCD1
ACETYLATUBULINLYS40
NDRG1_pT346
EPPK1
RB
HER3
CASPASE8
MYH11
ECADHERIN
ERALPHA
PARPCLEAVED
SNAIL
ERCC1
CKIT
BETACATENIN
EGFR_pY1173
BCLXL
PKCALPHA
HER2
SETD2
SMAC
CASPASE7CLEAVEDD198
IGFBP2
CMET
BAK
PAXILLIN
Unbalanced process 10
ASNS
SETD2
NDRG1_pT346
FASN
RB
ERCC1
HSP70
CAVEOLIN1
MYH11
GAPDH
ECADHERIN
CASPASE8
PARPCLEAVED
BAK
ACC1
CMET
NFKBP65_pS536
SNAIL
SMAC
STAT5ALPHA
Unbalanced process 11
ACETYLATUBULINLYS40
SYK
ERALPHA
HSP70
ECADHERIN
LCK
SNAIL
NFKBP65_pS536
PARPCLEAVED
EPPK1
STAT5ALPHA
GAPDH
GSK3_pS9
BETACATENIN
SETD2
CASPASE8
CKIT
CMET
PEA15_pS116
X1433ZETA
ERCC1
MYH11
CASPASE7CLEAVEDD198
S6_pS235S236
Unbalanced process 12

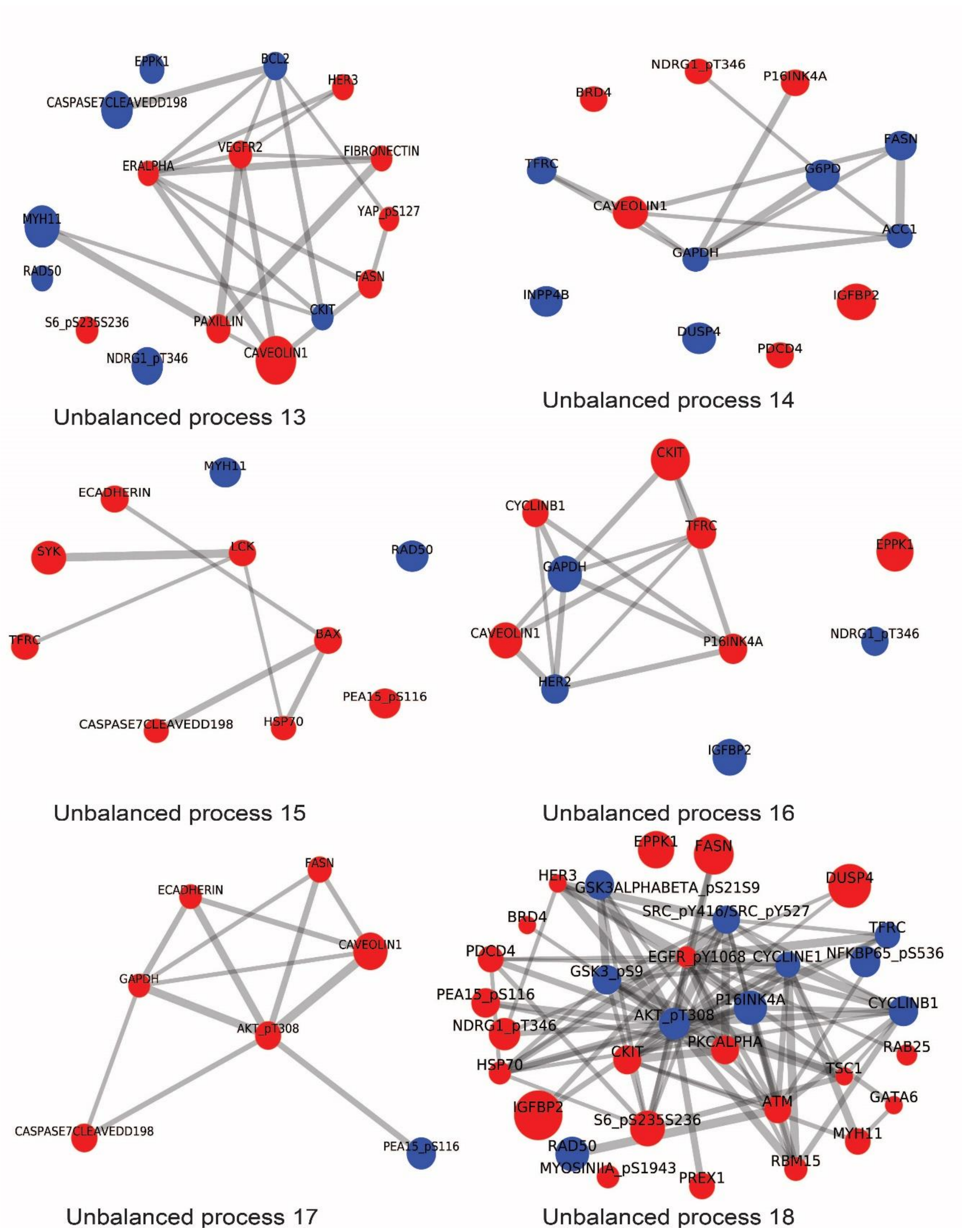
EPPK1
BCL2
HER3
CASPASE7CLEAVEDD198
VEGFR2
FIBRONECTIN
ERALPHA
YAP_pS127
MYH11
RAD50
FASN
S6_pS235S236
PAXILLIN
CKIT
NDRG1_pT346
CAVEOLIN1
Unbalanced process 13
NDRG1_pT346
P16INK4A
BRD4
FASN
TFRC
G6PD
CAVEOLIN1
ACC1
GAPDH
INPP4B
IGFBP2
DUSP4
PDCD4
Unbalanced process 14
MYH11
ECADHERIN
SYK
LCK
RAD50
TFRC
BAX
PEA15_pS116
CASPASE7CLEAVEDD198
HSP70
Unbalanced process 15
CKIT
CYCLINB1
TFRC
EPPK1
GAPDH
CAVEOLIN1
P16INK4A
NDRG1_pT346
HER2
IGFBP2
Unbalanced process 16
FASN
ECADHERIN
CAVEOLIN1
GAPDH
AKT_pT308
CASPASE7CLEAVEDD198
PEA15_pS116
Unbalanced process 17
EPPK1
FASN
HER3
GSK3ALPHABETA_pS21S9
DUSP4
BRD4
SRC_pY416/SRC_pY527
TFRC
PDCD4
EGFR_pY1068
CYCLINE1
NFKBP65_pS536
GSK3_pS9
PEA15_pS116
P16INK4A
CYCLINB1
AKT_pT308
NDRG1_pT346
PKCALPHA
RAB25
HSP70
CKIT
TSC1
GATA6
IGFBP2
ATM
S6_pS235S236
MYH11
RAD50
MYOSINIIA_pS1943
RBM15
PREX1
Unbalanced process 18

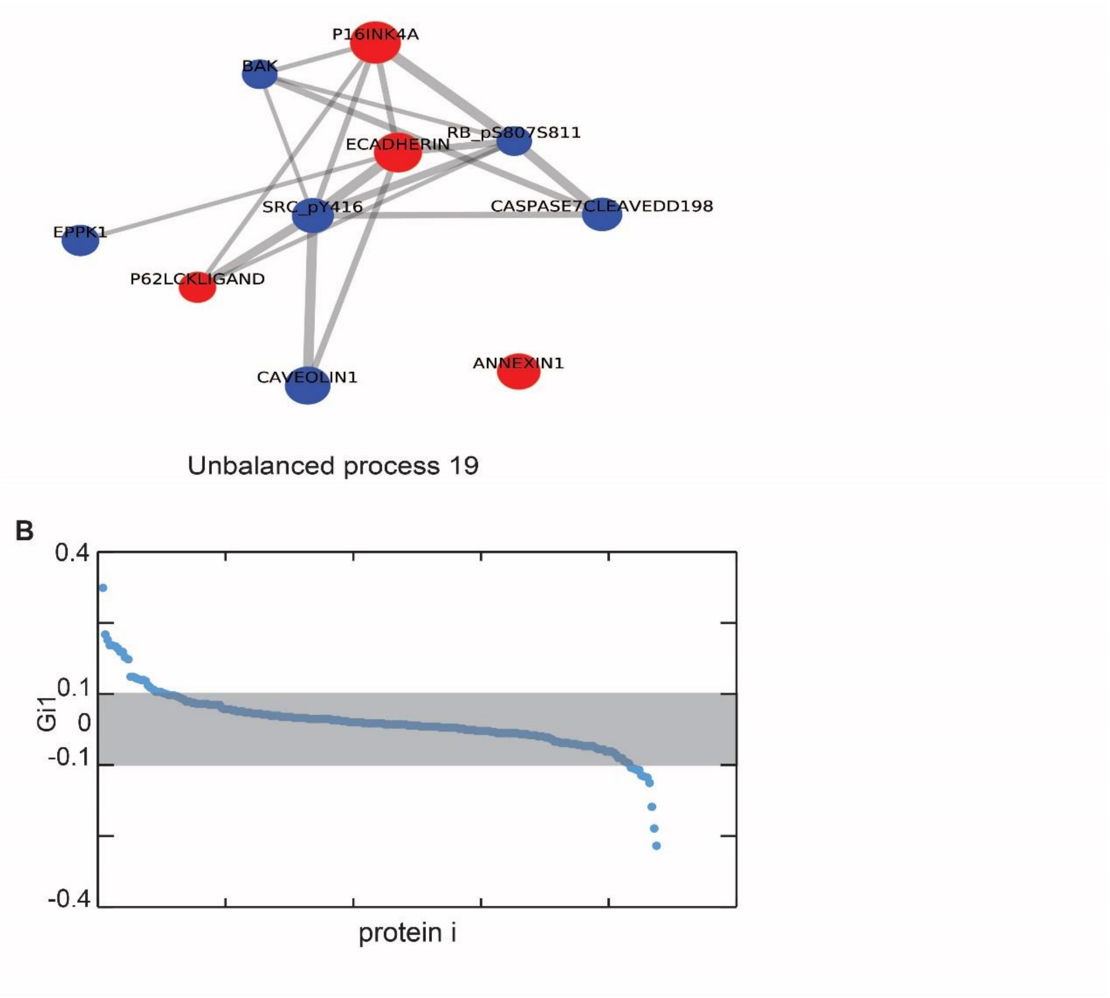

Figure S2. **19 unbalanced subnetworks, as identified by surprisal analysis, found to be active in 1038 tumors.** (**A**) 19 unbalanced processes (subnetworks) characterizing 1038 patients are shown. See Figure S3 demonstrating how the number of processes was determined. Proteins with negative Giα values are labeled in blue and proteins with positive Giα values in red, to distinguish between *correlated and anti-correlated proteins*. Circle sizes, denoting participating proteins in the process, are defined by Giα values. The procedure calculating induction or reduction in expression levels includes calculation of the product, $G_{i\alpha}\lambda_{\alpha}(k)$, which denotes a deviation in expression levels from the reference state due to process α. Positive values of $G_{i\alpha}\lambda_{\alpha}(k)$ mean induction relative to the steady state, and negative values mean reduction. For more details see references [1–3]. (**B**) Proteins must have a significant Giα (which represent the degree of participation of every protein *i* in the unbalanced process α, see Methods for more details) values to be included in the unbalanced process α. The proteins that take part in the different unbalanced processes were identified as follows: for every unbalanced process α, Giα values were sorted according to their size, and only proteins (*i*) with significant G*i*α values were considered to participate in the unbalanced process α. This is exemplified for the process α = 1. In the figure sorted values of G*i*1 are shown. The gray box represents threshold values. Proteins with G*i*1 > 0.1 or G*i*1 < -0. 1 (which form the top and bottom "tails" of the distribution) were considered to participate the most in the unbalanced process α = 1. These proteins were used to build a functional subnetwork using STRING database (showed in **A**).

Figure S3

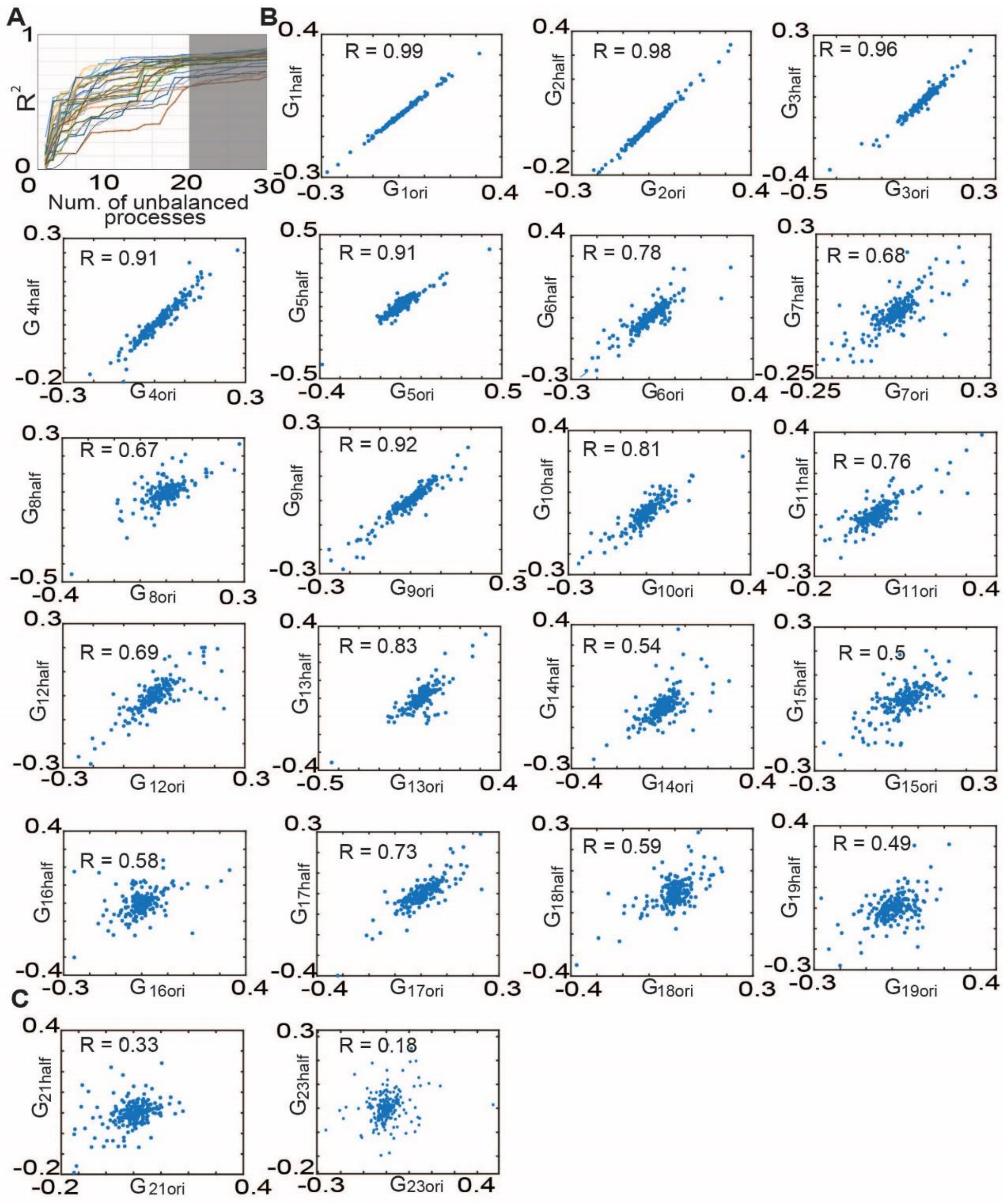

Figure S3. **19 unbalanced processes reproduce the experimental data.** (**A**) R^2 values, reflecting the correlation between the experimental expression data and the theoretical calculations, were calculated in order to determine how many processes are required in order to reproduce the experimental data. As we add more unbalanced processes we expect to get higher correlation between the experimental data and the theoretical calculation, reaching the highest level of correlation when the experimental data is reproduced by the theoretical fitting. When the R^2 values reach a plateau, that means that we do not add real processes, but mainly noise. Correlation plots between theoretical calculations and the experimental data are shown for 30 selected patients, harboring different types of cancer. R^2 was calculated by plotting the natural logarithm of the experimental data (LnXi) vs $\sum G_{i\alpha}\lambda_\alpha(k)$ for α = 1-30 (denoted as

Num. of unbalanced processes in the plot). $G_{i\alpha}$ denotes weight of protein *i* in a process α. $\lambda_\alpha(k)$ denotes amplitude of a process α in each patient *k*. Product, $G_{i\alpha}\lambda_\alpha(k)$, denotes a deviation in expression levels from the reference state due to process α. $\sum G_{i\alpha}\lambda_\alpha(k)$ is the sum of all the processes in which each protein participates (For more details see [1–3]). The plot reaches a plateau after the 19th process. This indicates that after the 19th process the data includes mainly noise. (**B**) Unbalanced processes remained the same when either the entire dataset or a subset of the dataset are used for the analysis. The weights of the proteins in the significant unbalanced processes (α = 1-19) were found to be similar when either the entire dataset (original (ori) dataset, 1038 patients) or only half the population of patients from each dataset (521 patients) were analyzed as indicated by the high correlation of the scatter plots. The correlation coefficient R is above 0.9 for the processes 0,1,2,3,4,5,and 9 and is above 0.65 for the processes 6,7,8,10,11,12,13 and 17. (**C**) The correlation coefficient R was significantly lower when the unbalanced processes 21, 23 were compared indicating that those processes represent mainly noise.

Figure S4

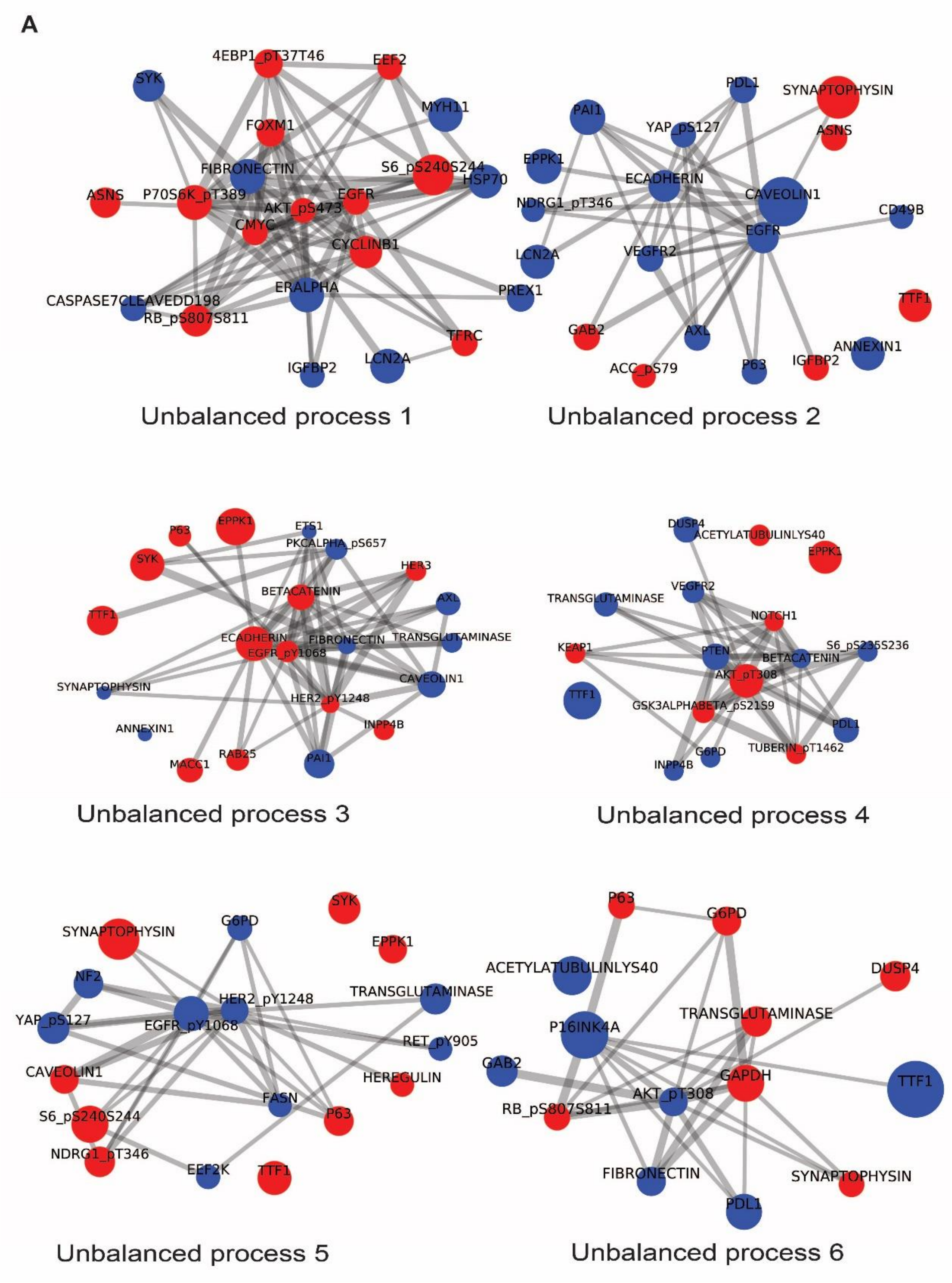
A
4EBP1_pT37T46
EEF2
SYK
MYH11
FOXM1
FIBRONECTIN
S6_pS240S244
HSP70
ASNS
P70S6K_pT389
EGFR
AKT_pS473
CMYC
CYCLINB1
CASPASE7CLEAVEDD198
ERALPHA
PREX1
RB_pS807S811
TFRC
IGFBP2
LCN2A
Unbalanced process 1
PDL1
SYNAPTOPHYSIN
PAI1
YAP_pS127
ASNS
EPPK1
ECADHERIN
CAVEOLIN1
NDRG1_pT346
CD49B
EGFR
LCN2A
VEGFR2
TTF1
GAB2
AXL
ANNEXIN1
ACC_pS79
P63
IGFBP2
Unbalanced process 2
EPPK1
ETS1
P63
PKCALPHA_pS657
SYK
HER3
BETACATENIN
TTF1
AXL
ECADHERIN
FIBRONECTIN
TRANSGLUTAMINASE
EGFR_pY1068
CAVEOLIN1
SYNAPTOPHYSIN
HER2_pY1248
INPP4B
ANNEXIN1
RAB25
PAI1
MACC1
Unbalanced process 3
DUSP4
ACETYLATUBULINLYS40
EPPK1
VEGFR2
TRANSGLUTAMINASE
NOTCH1
KEAP1
S6_pS235S236
PTEN
BETACATENIN
AKT_pT308
TTF1
GSK3ALPHABETA_pS21S9
PDL1
G6PD
TUBERIN_pT1462
INPP4B
Unbalanced process 4
SYK
SYNAPTOPHYSIN
G6PD
EPPK1
NF2
HER2_pY1248
TRANSGLUTAMINASE
YAP_pS127
EGFR_pY1068
RET_pY905
CAVEOLIN1
HEREGULIN
FASN
S6_pS240S244
P63
NDRG1_pT346
EEF2K
TTF1
Unbalanced process 5
P63
G6PD
ACETYLATUBULINLYS40
DUSP4
TRANSGLUTAMINASE
P16INK4A
GAB2
GAPDH
TTF1
AKT_pT308
RB_pS807S811
FIBRONECTIN
SYNAPTOPHYSIN
PDL1
Unbalanced process 6

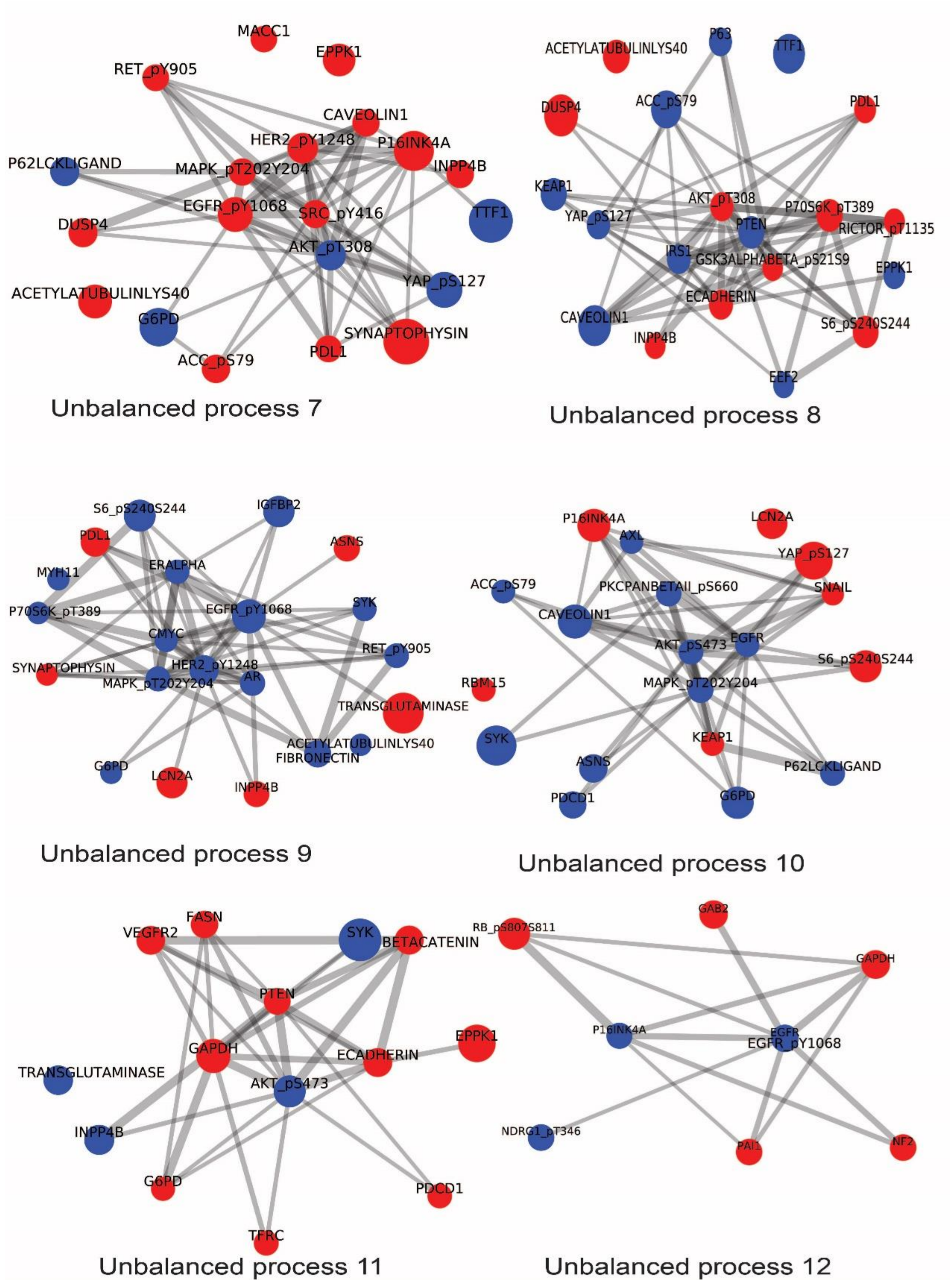
MACC1
EPPK1
RET_pY905
CAVEOLIN1
HER2_pY1248
P16INK4A
P62LCKLIGAND
MAPK_pT202Y204
INPP4B
EGFR_pY1068
SRC_pY416
TTF1
DUSP4
AKT_pT308
YAP_pS127
ACETYLATUBULINLYS40
G6PD
SYNAPTOPHYSIN
PDL1
ACC_pS79
Unbalanced process 7
ACETYLATUBULINLYS40
P63
TTF1
DUSP4
ACC_pS79
PDL1
KEAP1
AKT_pT308
P70S6K_pT389
YAP_pS127
PTEN
RICTOR_pT1135
IRS1
GSK3ALPHABETA_pS21S9
EPPK1
ECADHERIN
CAVEOLIN1
INPP4B
S6_pS240S244
EEF2
Unbalanced process 8
S6_pS240S244
IGFBP2
PDL1
ASNS
MYH11
ERALPHA
P70S6K_pT389
EGFR_pY1068
SYK
CMYC
RET_pY905
SYNAPTOPHYSIN
HER2_pY1248
MAPK_pT202Y204
AR
TRANSGLUTAMINASE
ACETYLATUBULINLYS40
FIBRONECTIN
G6PD
LCN2A
INPP4B
Unbalanced process 9
P16INK4A
LCN2A
AXL
YAP_pS127
ACC_pS79
PKCPANBETAII_pS660
SNAIL
CAVEOLIN1
AKT_pS473
EGFR
S6_pS240S244
RBM15
MAPK_pT202Y204
SYK
KEAP1
ASNS
P62LCKLIGAND
PDCD1
G6PD
Unbalanced process 10
FASN
VEGFR2
SYK
BETACATENIN
PTEN
EPPK1
GAPDH
ECADHERIN
TRANSGLUTAMINASE
AKT_pS473
INPP4B
G6PD
PDCD1
TFRC
Unbalanced process 11
GAB2
RB_pS807S811
GAPDH
P16INK4A
EGFR
EGFR_pY1068
NDRG1_pT346
PAI1
NF2
Unbalanced process 12

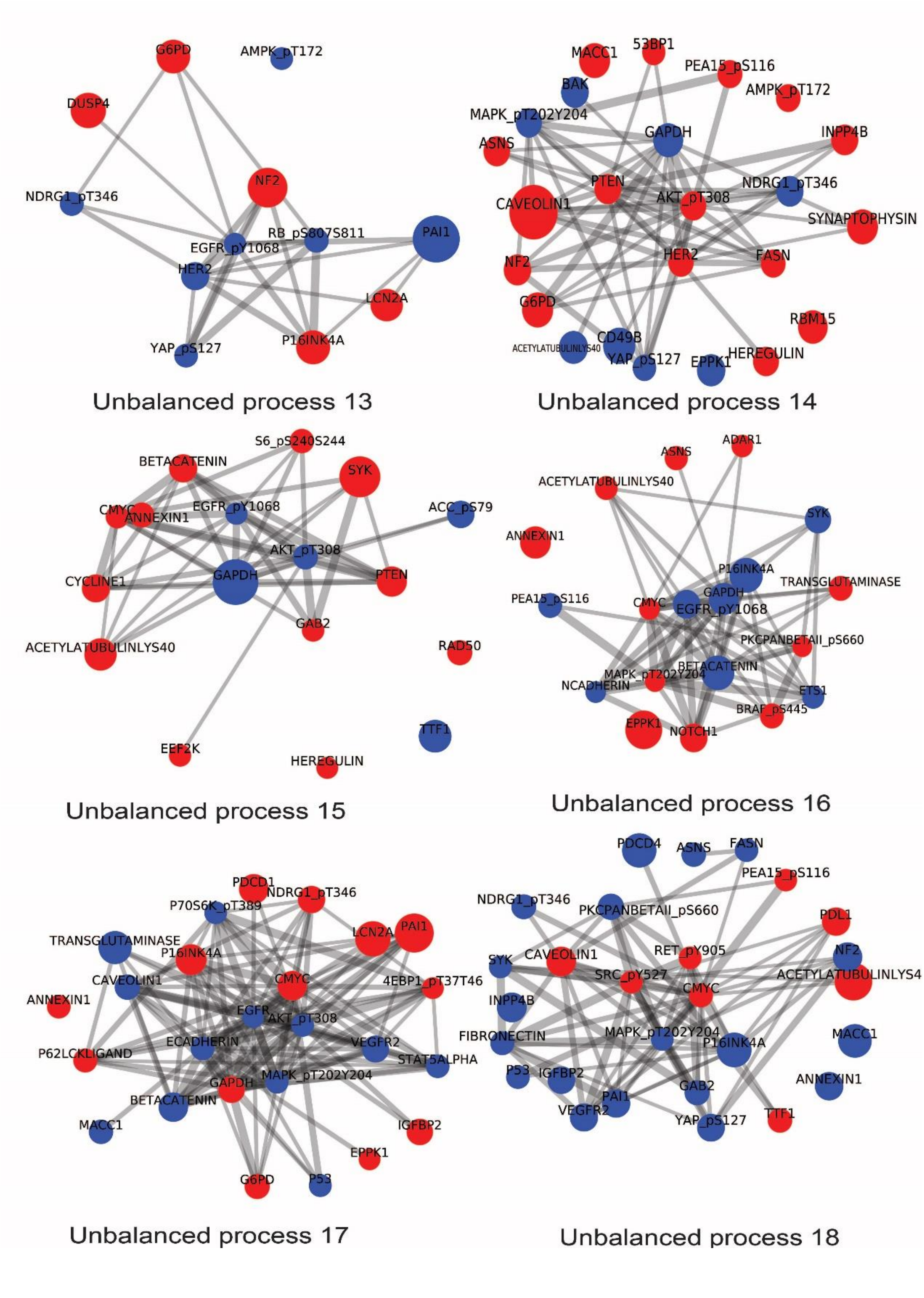
G6PD
AMPK_pT172
DUSP4
NF2
NDRG1_pT346
RB_pS807S811
PAI1
EGFR_pY1068
HER2
LCN2A
YAP_pS127
P16INK4A
Unbalanced process 13
MACC1
53BP1
PEA15_pS116
BAK
AMPK_pT172
MAPK_pT202Y204
GAPDH
INPP4B
ASNS
PTEN
NDRG1_pT346
CAVEOLIN1
AKT_pT308
SYNAPTOPHYSIN
NF2
HER2
FASN
G6PD
RBM15
CD49B
ACETYLATUBULINLYS40
YAP_pS127
EPPK1
HEREGULIN
Unbalanced process 14
S6_pS240S244
BETACATENIN
SYK
CMYC
ANNEXIN1
EGFR_pY1068
ACC_pS79
AKT_pT308
CYCLINE1
GAPDH
PTEN
GAB2
ACETYLATUBULINLYS40
RAD50
TTF1
EEF2K
HEREGULIN
Unbalanced process 15
ASNS
ADAR1
ACETYLATUBULINLYS40
SYK
ANNEXIN1
P16INK4A
GAPDH
TRANSGLUTAMINASE
PEA15_pS116
CMYC
EGFR_pY1068
PKCPANBETAII_pS660
MAPK_pT202Y204
BETACATENIN
NCADHERIN
ETS1
BRAF_pS445
EPPK1
NOTCH1
Unbalanced process 16
PDCD1
NDRG1_pT346
P70S6K_pT389
LCN2A
PAI1
TRANSGLUTAMINASE
P16INK4A
CAVEOLIN1
CMYC
4EBP1_pT37T46
ANNEXIN1
EGFR
AKT_pT308
ECADHERIN
VEGFR2
P62LCKLIGAND
STAT5ALPHA
GAPDH
MAPK_pT202Y204
BETACATENIN
MACC1
IGFBP2
EPPK1
G6PD
P53
Unbalanced process 17
PDCD4
ASNS
FASN
PEA15_pS116
NDRG1_pT346
PKCPANBETAII_pS660
PDL1
RET_pY905
NF2
SYK
CAVEOLIN1
SRC_pY527
CMYC
ACETYLATUBULINLYS4
INPP4B
FIBRONECTIN
MAPK_pT202Y204
P16INK4A
MACC1
P53
IGFBP2
GAB2
ANNEXIN1
PAI1
VEGFR2
YAP_pS127
TTF1
Unbalanced process 18

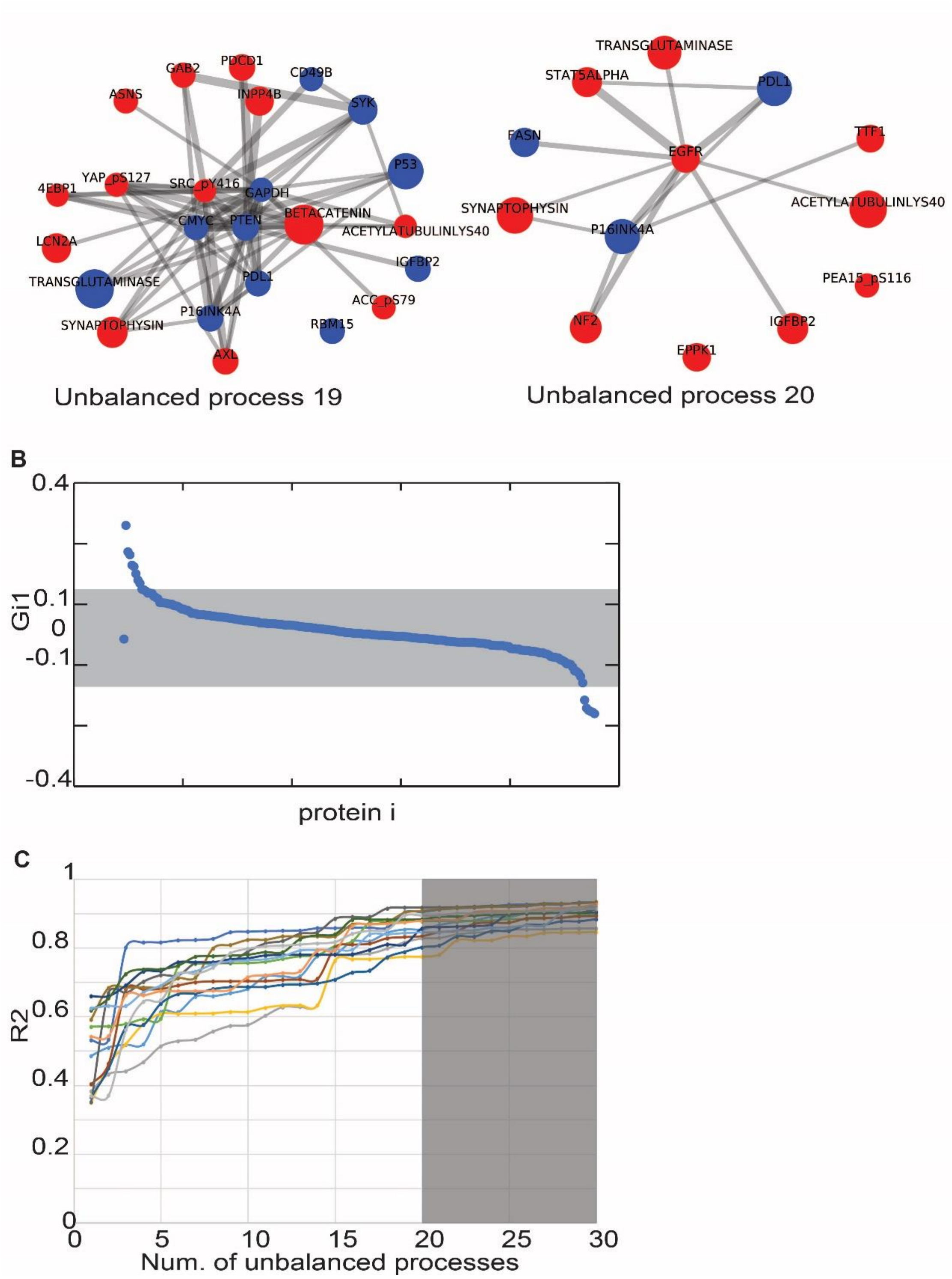

Figure S4. 20 unbalanced subnetworks, as identified by surprisal analysis, found to be active in 162 cancer cell lines. (**A**) 20 unbalanced processes characterizing 162 cancer cell lines. See Figure S4C demonstrating how the number of processes was determined. Proteins with negative Giα values are labeled in blue and proteins with positive Giα values in red, to distinguish between correlated and anti-correlated proteins. 20 unbalanced processes reproduce the experimental data. (**B**) Proteins must have a significant Giα (which represent the degree of

participation of every protein *i* in the unbalanced process α, see Methods for more details) values to be included in the unbalanced process α. The proteins that take part in the different unbalanced processes were identified as follows: For every unbalanced process α, Giα values were sorted according to their size, and only proteins with significant Giα values were considered to participate in the unbalanced process α. This is exemplified for the process α = 1. In the figure sorted values of Gi1 are shown. The gray box represents threshold values. Proteins with Gi1 > 0.15 or Gi1 < -0. 15 (which form the top and bottom "tails" of the distribution) were considered to participate the most in the unbalanced process α = 1. These proteins were used to build a functional subnetwork using STRING database (showed in **A**). (**C**) R^2 values for 14 selected cell lines, are shown as an example. The R^2 was calculated by plotting the natural logarithm of the experimental data (LnXi) vs $\sum G_{i\alpha}\lambda_{\alpha}(k)$ for α = 1-30. (see Figure legend S3 for more details). As we add more unbalanced processes, we expect to get higher correlation between the experimental data and the theoretical calculation ($\sum G_{i\alpha}\lambda_{\alpha}(k)$), reaching the highest level of correlation when the experimental data is reproduced by the theoretical fitting. The plot reaches a plateau after the 20th process. This indicates that after the 20th process the data includes mainly noise.

Figure S5

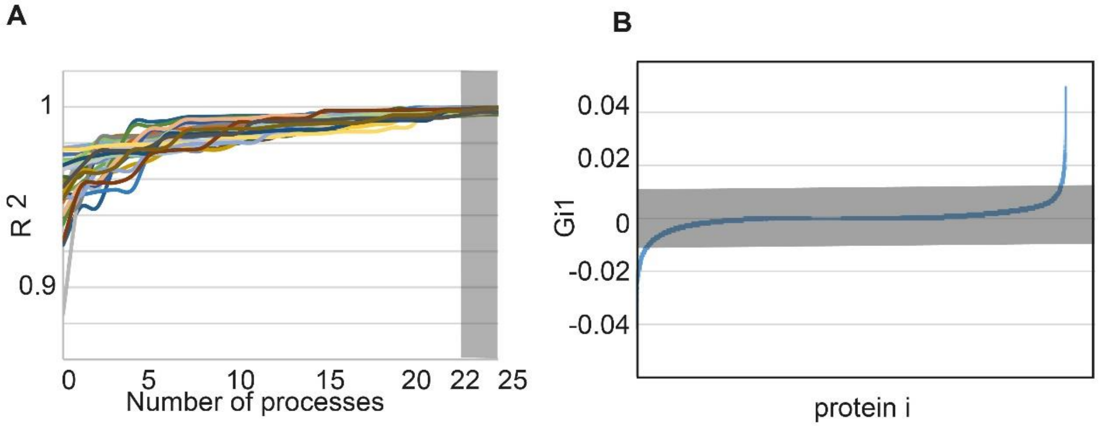

Figure S5. 22 unbalanced processes found to be active in 15 patient-derived HNSCC tissues treated with anti-EGFR monotherapy (**A**) R^2 values were calculated for all patients by plotting the natural logarithm of the experimental data against the sum of the gene expression level alterations due to unbalanced processes for different values of α. The figure shows that the plots for all patients reach a plateau after 22 processes, suggesting that the 22 unbalanced processes are significant, and the rest of the processes represent random noise in the samples. (**B**) The transcripts that take part in the different unbalanced processes were identified as follows: For every unbalanced process α, Giα values were sorted according to their size, and only transcripts with significant Giα values were considered to participate in the unbalanced process α. This is exemplified for the process α = 1 in the figure. Shown are sorted values of Gi_1, which represent the degree of participation of every transcript *i* in the unbalanced process α = 1. The gray box represents threshold values. Transcripts with $Gi_1 > 0.015$ or Gi1 < -0.015 (which are not contained in the gray box and form the top and bottom "tails" of the distribution) were considered to participate the most in the unbalanced process α = 1. These transcripts were used to find a biological meaning of each unbalanced processes using David database.

References:

1. Remacle F, Kravchenko-Balasha N, Levitzki A, Levine RD. Information-theoretic analysis of phenotype changes in early stages of carcinogenesis. Proc Natl Acad Sci U S A. 2010;107(22):10324–9.

2. Vasudevan S, Flashner-Abramson E, Remacle F, Levine RD, Kravchenko-Balasha N. Personalized disease signatures through information-theoretic compaction of big cancer data. Proc Natl Acad Sci U S A [Internet]. 2018 Jul 24 [cited 2019 Aug 8];115(30):7694–9. Available from: http://www.ncbi.nlm.nih.gov/pubmed/29976841

3. Flashner-Abramson E, Vasudevan S, Adejumobi IA, Sonnenblick A, Kravchenko-Balasha N. Decoding cancer heterogeneity: Studying patient-specific signaling signatures towards personalized cancer therapy. Theranostics. 2019;9(18):5149–65.
